# Myeloid-T cell proximity is prominent in healthy pregnancies with extreme fetal-maternal HLA incompatibility

**DOI:** 10.1016/j.isci.2025.114179

**Published:** 2025-11-21

**Authors:** Xuezi Tian, Juliette Krop, Marieke E. Ijsselsteijn, Johanna M. Kapsenberg, Jacqueline D.H. Anholts, Lotte van der Meeren, Hailiang Mei, Michiel H.J. Huigen, Carin van der Keur, Dave L. Roelen, Lisa E.E.L.O. Lashley, Els van Beelen, Frits Koning, Marie-Louise P. van der Hoorn, Michael Eikmans

**Affiliations:** 1 Department of Immunology, Leiden University Medical Center, Leiden, the Netherlands; 2 Department of Obstetrics and Gynecology, Leiden University Medical Center, Leiden, the Netherlands; 3 Department of Pathology, Leiden University Medical Center, Leiden, the Netherlands; 4 Department of Pathology, Erasmus Medical Center, Rotterdam, the Netherlands; 5 Department of Biomedical Data Sciences, Leiden University Medical Center, Leiden, the Netherlands

**Keywords:** immunology, pregnancy, omics

## Abstract

Pregnancy requires local immune tolerization. Oocyte donation (OD) pregnancies, with extensive fetal-maternal human leukocyte antigen (HLA) mismatching, are at higher risk of pre-eclampsia. We hypothesize that immune adaptations are needed at the fetal-maternal interface to maintain healthy pregnancy despite high HLA dissimilarity. By multispectral imaging, myeloid cells constituted 65% of the decidual immune cells and encompassed 12 distinct clusters. Fully-allogeneic healthy OD pregnancies displayed a higher frequency of CD163^+^HLA-DR^+^ myeloid cells and FoxP3^+^CD4^+^ Tregs near CD4^+^ T cells compared to semi-allogeneic healthy pregnancies and pre-eclampsia, together with a Treg-reinforcing gene signature. Contrastingly, pre-eclampsia was characterized by enhanced inflammatory chemokine expression and oxidative-stress-gene imbalance. Pregnancy outcomes were unaffected by decidual pathology, maternal HLA antibodies, or fetal HLA-C/maternal KIR haplotypes. This study highlights the frequency, phenotypic diversity, and T cell proximity of decidual myeloid cells in OD pregnancies and suggests their immune regulatory effects to compensate for higher fetal-maternal HLA mismatch loads.

## Highlights

• Decidual myeloid cells dominate immune landscape in oocyte donation pregnancies
• Myeloid-T cell proximity reflects tolerance in high HLA-mismatch OD pregnancies
• Healthy OD pregnancies display Treg gene signature and reduced inflammation
• Pre-eclamptic OD pregnancies show inflammatory and oxidative stress dysregulation

## Graphical abstract

**Figure:**
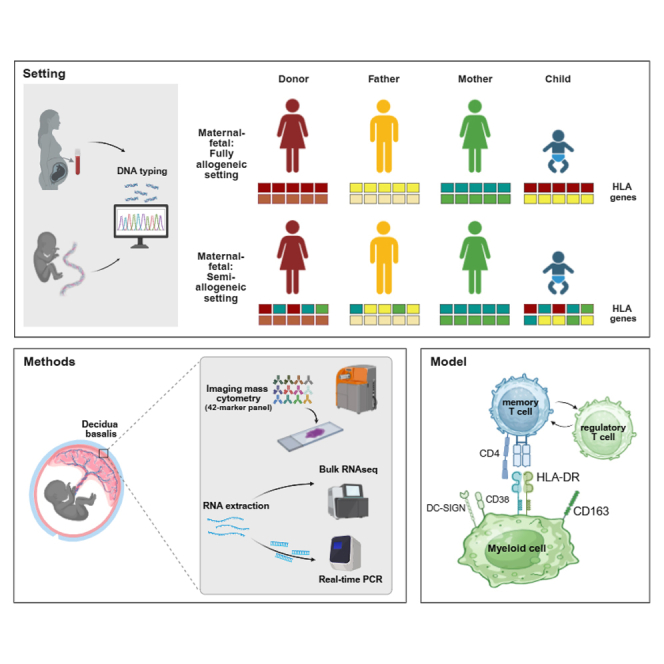

## Introduction

In naturally conceived (autologous) pregnancy, the fetus is essentially a semi-allograft carrying both maternal and paternal antigens. To maintain healthy pregnancy, the fetal-maternal interface (the decidua basalis) reflects a tolerogenic environment.^1^^,^^2^ Oocyte donation (OD) has been widely used over the last decades, amounting to 8% of all assisted reproductive technology treatment cycles in European countries.^3^ Compared with autologous oocyte *in vitro* fertilization (IVF) pregnancy, OD pregnancy is related to a higher degree of fetal-maternal immunogenetic dissimilarity, as both the oocyte and the sperm are from allogeneic individuals in relation to the gestational carrier. These allogeneic fetal cells encounter maternal immune cells after implantation at the decidua basalis, which makes OD pregnancy an interesting model for understanding maternal immune tolerance toward the fetus.^4^ OD pregnancies are related to a higher incidence of pregnancy complications, including pre-eclampsia (PE),^5^^,^^6^ partially because the pathogenesis of PE is linked to local immune responses.^7^^,^^8^ Hence, the high extent of fetal-maternal human leukocyte antigen (HLA) dissimilarity and accompanying immune reaction is expected to influence the outcome of OD pregnancies.

Macrophages and other myeloid cells remain consistently present in the decidua throughout the entire duration of pregnancy, playing a crucial role at the fetal-maternal interface.^9^ Macrophage populations are frequently categorized into two subsets and denominated as M1 and M2. The former are considered as inflammatory types, while M2 macrophages are responsible for immune tolerization.^10^ However, an increasing amount of research in recent years recognized the myeloid cell lineage as a more heterogeneous population and indicated that macrophages exhibit remarkable plasticity, with their characteristics constantly changing in response to signals from the surrounding environment.^11^^,^^12^^,^^13^ In line with this plasticity, studies in healthy pregnancies have shown that decidual macrophages cannot be strictly classified into the conventional M1/M2 categorization but they exhibit both proinflammatory and anti-inflammatory capacities.^14^ An in-depth phenotypic analysis for visualizing distinct macrophage subtypes has not yet been performed in the decidua of OD pregnancies.

*In situ* techniques like imaging mass cytometry (IMC) and multispectral immunofluorescence (IF) provide the possibility to study frequency and spatial distribution of decidual immune cell subsets, thereby avoiding enzymatic isolation processes and possible loss of cell types. During gestation of autologous pregnancy, our group showed that decidual myeloid cells represent the most dominant cell type in the microenvironment of trophoblasts and other maternal immune cells.^15^ As the main type of antigen-presenting cells, decidual macrophages have been described to express HLA-DR.^9^^,^^15^^,^^16^ This expression would enable communication with CD4^+^ T helper cells, thereby shaping local immune responses.

Fortunately, the majority of OD pregnancies remain healthy until term. This may consequently require that the maternal immune system exerts an even higher extent of tolerization than in autologous pregnancies. Consistent with this, decidual immune cells in placentas after OD tend to display immune regulatory phenotypes. A distinct infiltrate with high expression of CD14 (a general marker for macrophages) and CD163 (specific for M2 macrophages) was found in the chorionic plate of healthy OD pregnancies.^17^ Previous studies from our group showed that healthy OD pregnancies with a high extent of fetal-maternal HLA mismatches display a higher frequency of FoxP3^+^ Tregs^18^ and CD163^+^/CD14^+^ ratio in the decidua, compared to autologous pregnancies.^19^

In the current study, we hypothesized that in pregnancies with extreme fetal-maternal HLA mismatches leading to healthy outcomes, decidual immune cells create a more immune-regulatory microenvironment. Conversely, pregnancies lacking such a microenvironment might face the risk of PE. Utilizing advanced *in situ* immunostaining methods, we analyzed decidua cell types and their microenvironment across various pregnancy conditions, aiming to uncover the pathogenesis that influences the outcomes of OD pregnancies. We investigated the spatial microenvironment of decidual myeloid cells and T cells and employed RNA sequencing to assess subsequent molecular alterations. Our analyses support the notion that robust immune regulation is essential for successful OD pregnancy in the context of high HLA dissimilarity.

## Results

### Decidual myeloid cells exhibit the highest frequency among all the decidual immune cells

We applied a 42-marker IMC panel (Table S1) to assess the frequency of decidual immune cells and trophoblasts, as well as their spatial composition in healthy OD pregnancies. Among the eight samples selected for IMC staining, fully-allogeneic and semi-allogeneic groups did not significantly differ in any of the clinical characteristics (Table 1).

**Table 1:** Characteristics of IMC samples

Clinical parameter	Fully-allogeneic OD-healthy (*n* = 4)	Semi-allogeneic OD-healthy (*n* = 4)
**Mother**		

Age (year)	42 (37–47)	43 (39–47)
GA (weeks)	38.9 (38.7–39.1)	39.6 (39.0–40.0)
G	3 (2–6)	3 (1–5)
P	0.5 (0–1)	0 (0–2)
Miscarriage	1.5 (0–4)	0 (0–2)
Highest diastole (mmHg)	70 (60–80)	80 (80–83)
Fetal-maternal HLA mismatches (4-digit)	14 (11–16)	8 (5–9)∗
HLA-A mismatches	1.5 (1–2)	1 (0–1)
HLA-B mismatches	2 (2–2)	1.5 (1–2)
HLA-C mismatches	2 (2–2)	1 (1–2)
HLA-DR mismatches	3.5 (3–4)	2 (0–2)∗
HLA-DQ mismatches	3.5 (2–4)	1.5 (0–2)∗
HLA-DP mismatches	1.5 (1–2)	1 (1–2)

**Child**		

Gender (male) Number (percentage)	1 (25%)	1 (25%)
Birth weight (g)	3,520 (3,080–3,925)	3,575 (3,145–3,910)

GA, gestation age; G, gravidity; P, parity.

**Figure 1:**
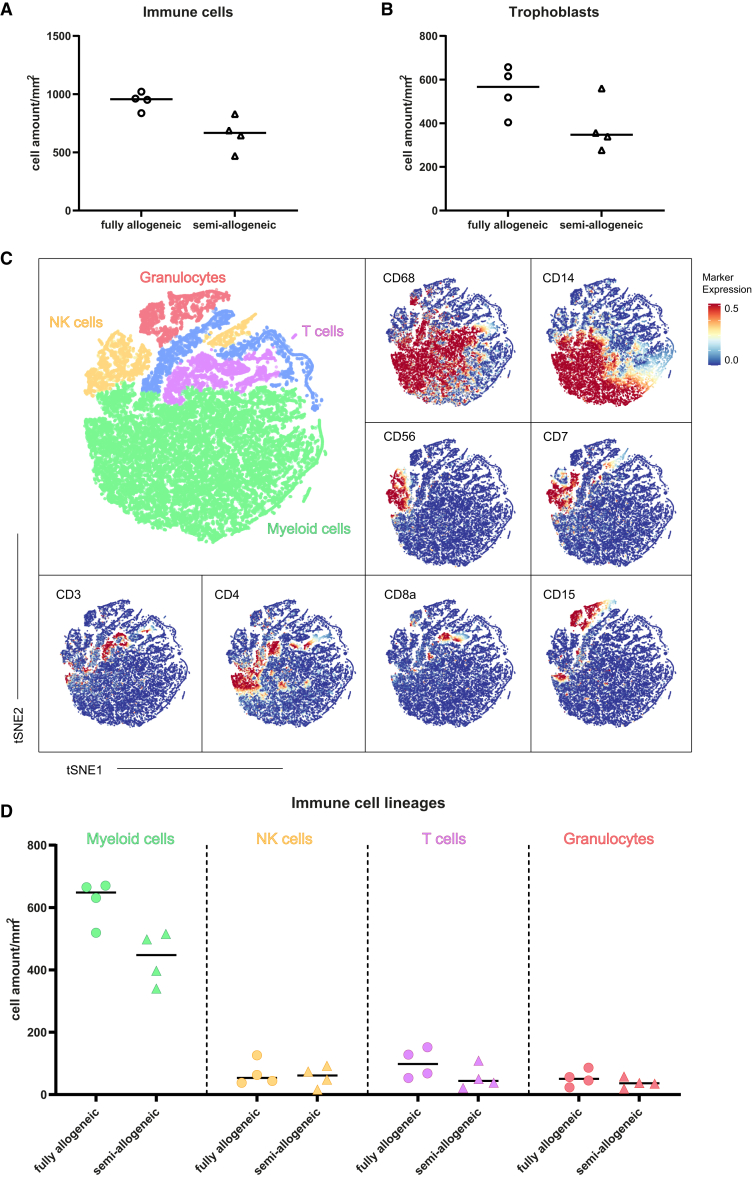
Comparison of major cell lineage frequencies at the maternal-fetal interface in OD-healthy samples using IMC (A and B) Immune cell and trophoblast cell amounts per mm^2^ basalis area in fully-allogeneic and semi-allogeneic groups. Data are shown as individual points, together with the median. (C) tSNE of the major immune cell lineages within the CD45^+^ compartment of IMC by automatic clustering using major immune cell lineage markers. The marker expression is binarized from 0 to 1, meaning one cell with 0.5 expression has 50% of pixels in the cell being positive for the marker. (D) Comparison of major immune cell lineages in decidua between fully-allogeneic (dots) and semi-allogeneic groups (triangles) using cell amount per mm^2^ basalis area. Data are shown as individual points, together with the median.

Decidual immune cells and trophoblasts were identifiable in each region of interest (ROI) and distinguished using lineage markers (CD45, CD14, CD3, CD7, and CD15) and trophoblast marker HLA-G, respectively (Figures S2A and S2C). No significant differences were observed in the total number of immune cells and trophoblasts per basalis area between the fully-allogeneic and semi-allogeneic OD-healthy groups (Figures 1A and 1B). ∗*p* < 0.05 (Chi-square test).

Several major immune cell lineages could be further identified within the total decidual immune cells, namely, myeloid cells (CD68^+^ and CD14^+^), T cells (CD3^+^, CD4^+^, or CD8a^+^), natural killer (NK) cells (CD56^+^ and CD7^+^), and granulocytes (CD15^+^) (Figures 1C and S2B). Although CD138^+^ plasma cells were present in the raw stained images (Figure S1D), their low numbers made it challenging to separate them from other immune cells in t-distributed stochastic neighbor embedding (tSNE) (blue-colored cell cluster in Figure 1C). Among the four major immune cell lineages, myeloid cells exhibited the highest cell amount per basalis area (420–640 cells/mm^2^) and the highest percentages in total immune cells (at least 65%). Myeloid cell frequencies were not significantly different between fully-allogeneic and semi-allogeneic OD-healthy groups and neither were frequencies of T cells, NK cells, and granulocytes (Figures 1D and S3A).

### Twelve phenotypically distinct clusters identified within the decidual myeloid cell lineage

**Figure 2:**
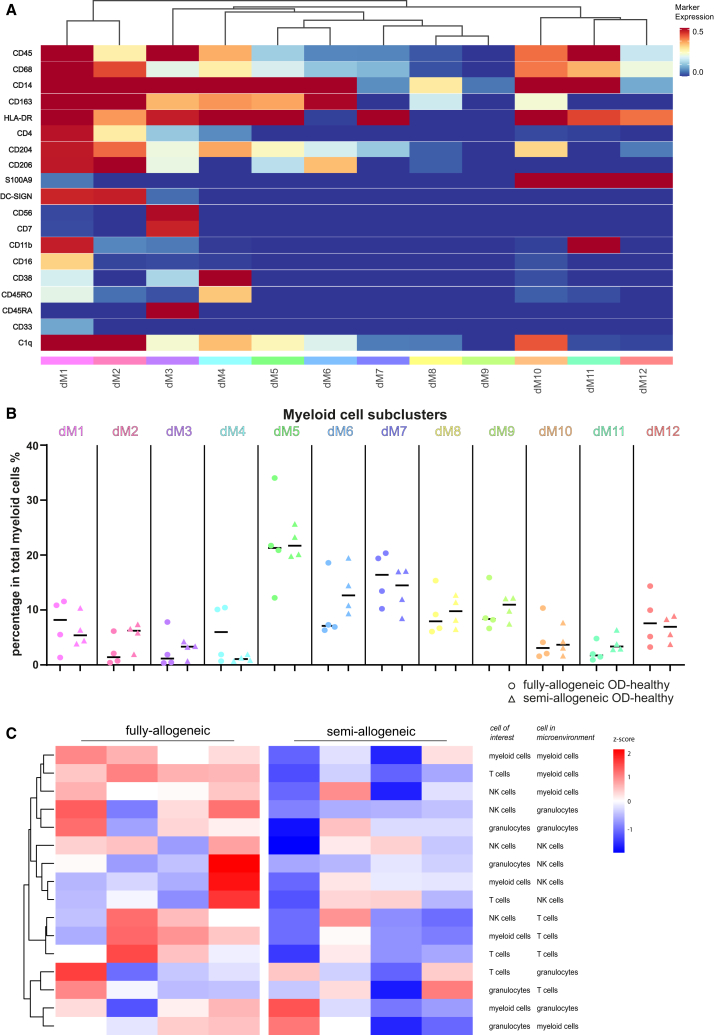
Comparison of myeloid cell clusters at the maternal-fetal interface in OD-healthy samples using IMC (A) Heatmap shows different myeloid-related marker expression patterns of 12 decidual myeloid clusters. The marker expression is binarized from 0 to 1, meaning one cell with 0.5 expression has 50% of pixels in the cell being positive for the marker. Subcluster names are displayed at the bottom of the heatmap; marker names are on the left. (B) Comparison of myeloid cell cluster percentages within total myeloid cells in decidua between fully-allogeneic (dots) and semi-allogeneic groups (triangles). Data are shown as individual points, together with the median. (C) *Z* score hierarchical clustering heatmap visualization of decidual immune cell lineage interactions with each other. Orange/red indicates a higher amount of interactions, and blue indicates a lower amount of interactions than the mean of corrected microenvironment analysis frequency per row.

Myeloid cells showed notable plasticity in the decidua of OD-healthy pregnancies. Utilizing 15 markers associated with myeloid cells, we identified 12 phenotypically distinct clusters within the myeloid cell compartment (Figures 2A, S2D, and S4A). Among these, eight clusters were CD163^+^ and four clusters were CD163^-^.

Within the decidual CD163^+^ myeloid cells, four clusters were HLA-DR^+^ and CD206^+^, with one cluster additionally expressing DC-SIGN, CD38, CD45RO, CD11b, and CD16 (dM1); another cluster was solely positive for DC-SIGN (dM2) and a third cluster had no clear positivity for additional markers (dM5). Two clusters within decidual CD163^+^ myeloid cells were positive for HLA-DR and negative for CD206, with one being CD38^+^ and CD45RO^+^ (dM4) and the other expressing S100A9 (dM10). Additionally, two clusters within CD163^+^ cells were HLA-DR^−^, with one cluster positive for CD206 and CD204 (dM6) and another negative for both markers (dM8). We also found one cluster that was positive for both myeloid cell markers and NK cell markers (CD56 and CD7) (dM3), which is also positive for CD38 and CD45RA. Investigation of this cluster in raw image data revealed primarily double-positive cells with a single nucleus, although some cells with merged nuclei (suggesting overlapping cells) were also observed. Due to the inability to separate nuclei in some cells, we compared CD163 and CD56 double-positive pixels within this myeloid cluster and found no significant difference between fully-allogeneic- and semi-allogeneic groups (data not shown).

In decidual CD163^−^ myeloid cells, three clusters were positive for HLA-DR. Among these, one cluster expressed CD204 but was negative for S100A9 (dM7). The other two clusters were positive for S100A9, with one also expressing CD11b (dM11) and the other displaying CD204 expression (dM12). Additionally, there was a cluster in decidual CD163^-^ myeloid cells that was only positive for CD14 (dM9). We incorporated CD11c in our antibody panel, as this marker has been used to distinguish and isolate a subset of macrophages.^14^ However, our *in situ* placental tissue analyses staining for CD11c gave non-specific results, which were difficult to interpret. Hence, we left out this marker from the results.

While myeloid clusters were observed in every sample, and with varying frequencies, there were no significant differences in percentages between the fully-allogeneic and semi-allogeneic OD-healthy group (Figure 2B).

T cells could be separated into CD4^+^ T cells, CD8^+^ T cells, and double-negative T cells (dnT cells). Their frequencies were comparable between groups. Further separation could not be performed for NK cells and granulocytes, as the type of markers included in the panel did not allow their separation into clusters that contained sufficient cell amounts.

### Fully-allogeneic OD-healthy group exhibits a high presence of decidual CD4^+^ T cells and CD163^+^HLA-DR^+^ myeloid cells in each other’s microenvironment

**Figure 3:**
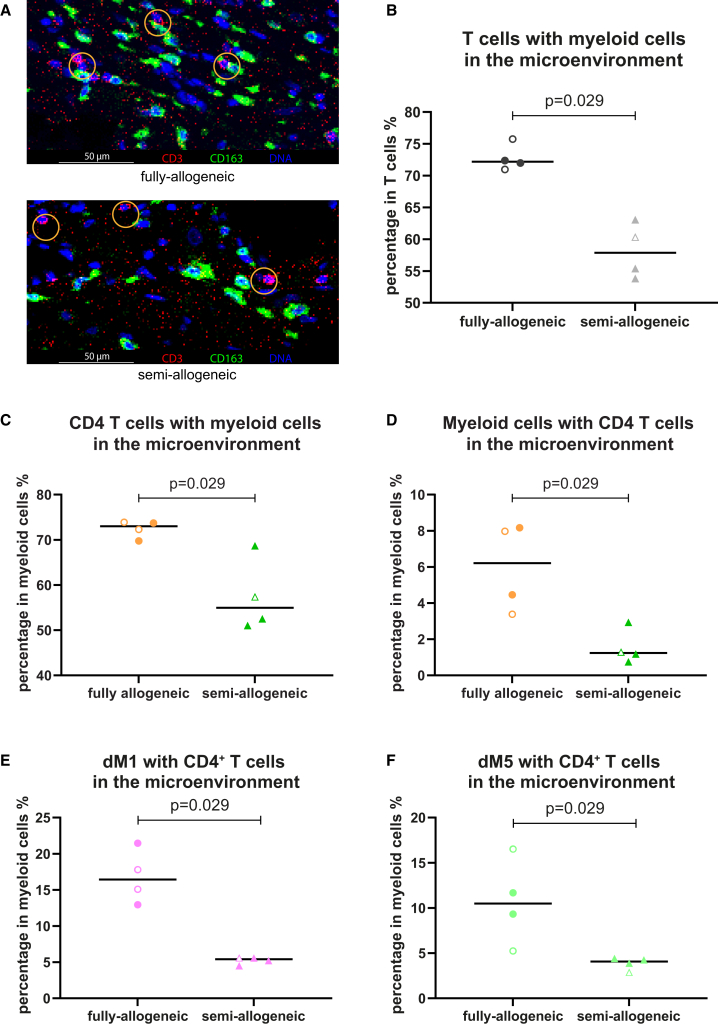
Decidual immune cell microenvironment in OD-healthy samples using IMC (A) Representative pictures of decidual myeloid cell and T cell microenvironments in fully-allogeneic and semi-allogeneic groups. Yellow circles demonstrate that in fully-allogeneic group, decidual myeloid cells and T cells have higher chances of being close to each other than in semi-allogeneic group. Scale bars, 50 μm. (B–F) Frequencies of the T cells/myeloid cells, including their subclusters, that are within the microenvironment of each other in fully-allogeneic and semi-allogeneic groups. Data were shown individually, together with the median. Mann-Whitney U tests were used to test the statistical difference; the *p* values are shown above the flags in the figures. Open dots represent parous women.

For spatial cell orientation we conducted analysis of the microenvironment (10-μm radius) surrounding all immune cell lineages, clusters, and trophoblasts in the decidua basalis of OD-healthy pregnancies. The fully-allogeneic OD-healthy group, in comparison to the semi-allogeneic OD-healthy group, has a higher frequency of decidual immune cells in each other’s microenvironment, although significant differences were only found in the microenvironment of T cells with myeloid cells (Figure 2C). Besides correcting for cell frequency, we also used the permutation test to confirm that these interactions did not occur at random (Figure S3B). Visualizing raw stained images for the immune cells, indeed, revealed a high frequency of T cells and myeloid cells in each other’s microenvironment especially in the fully-allogeneic group (Figure 3A). Statistical analysis confirmed the significant difference of abundance of T cells with myeloid cells in the microenvironment between the groups (*p* = 0.029, Figure 3B). We also analyzed trophoblasts and maternal immune cells in each other’s microenvironment, but no significant difference was found between groups (data not shown).

Building on the findings in immune lineages, we further analyzed the microenvironment of myeloid cell and T cell clusters. Myeloid cells and CD4^+^ T are more likely to be within each other’s microenvironment in the fully-allogeneic group compared to the semi-allogeneic group (*p* = 0.029, Figures 3C and 3D). Specifically, dM1 and dM5, which share positivity for CD163 and HLA-DR (Figure S2D), had a higher likelihood of containing CD4^+^ T cells in the microenvironment of the fully-allogeneic group compared to the semi-allogeneic group (*p* = 0.029, Figures 3E and 3F). The microenvironments of myeloid cell clusters that contain CD8^+^ T cells (Figures S4B and S4C), dnT cells, NK cells, or granulocytes did not show significant differences between the two groups (data not shown).

**Figure 4:**
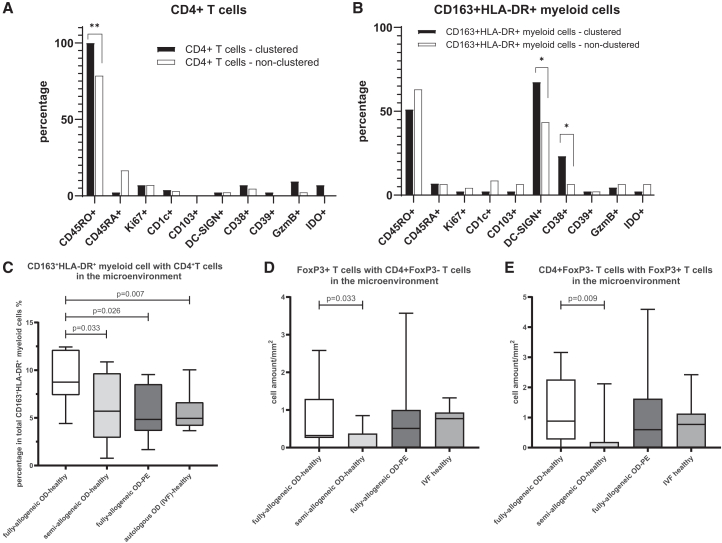
Microenvironment analysis of CD163^+^HLA-DR^+^ myeloid cells and CD4^+^ T cells (A and B) Frequencies of clustered/non-clustered decidual CD163^+^HLA-DR^+^ myeloid cells/CD4^+^ T cells that are positive for certain markers. Fisher’s exact tests were used to test the statistical difference between any two groups. Comparison with *p* values lower than 0.05 are indicated by ∗ and those lower than 0.01 by ∗∗. (C) Frequencies of the CD163^+^HLA-DR^+^ myeloid cells that are within the microenvironment of CD4^+^ T cells in four pregnancy groups (*n* = 36) using multispectral immunofluorescence. Data are represented as min to max boxplots. Mann-Whitney U tests were used to test the statistical difference between any two groups; significant *p* values (<0.05) are stated above the flags. (D and E) Frequencies of FoxP3^+^ T cells and CD4^+^FoxP3^−^ T cells within each other’s microenvironment. Since the cell amount of FoxP3^+^ T cells is relatively low in the decidua, the cell amount per mm^2^ is shown. Data are represented as min to max boxplots. Mann-Whitney U tests were used to test the statistical difference between groups; significant *p* values are shown above the flags.

We next compared marker expression between individual CD4^+^ T cells that were in the microenvironment of CD163^+^HLA-DR^+^ myeloid cells (“clustered”) and CD4^+^ T cells that were not in this microenvironment (“non-clustered”). The former were more often CD45RO^+^ (100.0% vs. 78.6%, *p* < 0.005), suggesting a memory phenotype (Figure 4A). We did the same for individual CD163^+^HLA-DR^+^ myeloid cells: the ones in the microenvironment of T cells more often were DC-SIGN^+^ (67.4% vs. 43.5%) and CD38^+^ (23.3% vs. 6.5%) than those not in this microenvironment (both *p* < 0.05, Figure 4B).

### CD163^+^HLA-DR^+^ myeloid cells with CD4^+^ T cells in the microenvironment are more often seen in fully-allogeneic OD-healthy group than in fully-allogeneic OD pregnancies with PE

To validate our findings from IMC data in a larger cohort and to further identify the phenotype of CD4^+^ T cells involved, we established a multispectral IF panel incorporating six markers (CD163, HLA-DR, CD3, CD4, FoxP3, and DAPI) (Table S2). Here, an OD group with PE and a non-donor group after IVF were included. The IF panel was applied to fully-allogeneic OD-healthy (*n* = 11), semi-allogeneic OD-healthy (*n* = 8), fully-allogeneic OD-PE (*n* = 8), and autologous OD (IVF)-healthy (*n* = 9). The fully-allogeneic OD-PE group exhibited significantly higher diastole, lower gestational age, and lower fetal birth weight compared to any other group (Table 2), aligning with the diagnosis and clinical presentation of PE.

**Table 2:** Characteristics of multispectral immunofluorescence samples

Clinical parameters	Fully-allogeneic OD-healthy (*n* = 11)	Semi-allogeneic OD-healthy (*n* = 8)	Fully-allogeneic OD-PE (*n* = 8)	IVF-healthy (*n* = 9)
**Mother**				

Age (year)	41 (28–48)	42 (35–47)	40 (30–44)	39 (32–41)^#^
GA (weeks)	39.0 (37.6–39.3)	38.9 (37.0–40.0)	36.7 (32.1–40.6)∗	39.6 (37.0–40.9)
G	3 (1–9)	2 (1–5)	1 (1–3)	2 (1–5)
P	1 (0–2)	0 (0–2)	0 (0–1)	0 (0–2)
Miscarriage	1 (0–6)	0 (0–3)	0 (0–1)	0 (0–4)
Highest diastole (mmHg)	80 (60–86)	80 (70–83)	95 (90–115)∗	85 (65–95)
Fetal-maternal HLA mismatches (4-digit)	13 (10–16)	7.5 (3–9) •	13 (10–16)	–
HLA-A mismatches	1 (0–2)	1 (0–1)	1 (0–2)	–
HLA-B mismatches	2 (1–2)	1 (0–2) •	2 (0–2)	–
HLA-C mismatches	2 (1–2)	1 (0–2) •	2 (1–2)	–
HLA-DR mismatches	3 (2–4)	2 (0–2) •	3.5 (2–4)	–
HLA-DQ mismatches	4 (2–4)	2 (0–2) •	3.5 (2–4)	–
HLA-DP mismatches	2 (1–2)	1 (0–3)	1.5 (0–2)	–

**Child**				

Gender (male) Number (percentage)	4 (36.4%)	3 (37.5%)	2 (25.0%)	5 (55.6%)
Birth weight (g)	3,710 (2,510–4,500)	3,575 (2,910–4130)	2,707 (1,600–3,450)∗	3,360 (2,710–4,080)

Categorical variable (gender) is described using numbers and percentages. Fisher’s exact tests were performed to determine *p* value, Other variables are all non-normally distributed numerical variables, which are described by median with the minimum and maximum. Mann-Whitney U tests were performed to determine *p* value. Data are marked with ∗ in the fully-allogeneic OD-PE group for having significant differences compared with any other three groups. Data are marked with # in the IVF-healthy group for having significant differences compared with semi-allogeneic OD-healthy group. Data are marked with • in the semi-allogeneic OD-healthy group for having significant differences compared with two fully-allogeneic OD groups. Other data show no significant difference between groups.

We identified CD163^+^HLA-DR^+^ myeloid cells and CD4^+^ T cells in each sample, whereby FoxP3^+^ Tregs could be distinguished in the CD4^+^ T cell population. Comparisons of cell amounts per basalis area showed no significant differences in the frequency of these three cell types between any of the groups (Figures S4B–S4D). To study immune cell microenvironments, we employed methods similar to those used in the IMC analysis. The extent of CD163^+^HLA-DR^+^ myeloid cells and CD4^+^ T cells in each other’s microenvironment was significantly higher in the fully-allogeneic OD-healthy group compared to the semi-allogeneic OD-healthy group (*p* = 0.033) as well as to the fully-allogeneic OD-PE group (*p* = 0.026) and autologous OD-healthy group (*p* = 0.007) (Figure 4C).

The multispectral IF allowed us to scan a larger tissue area, so that the number of FoxP3^+^ T cells per basalis area could be further identified. Frequencies of myeloid cells and FoxP3^+^ T cells in each other’s microenvironment were not different between groups. However, the amount of FoxP3^+^ T cells and other CD4^+^ (FoxP3^−^) T cells in each other’s microenvironment was significantly higher in the fully-allogeneic group than in the semi-allogeneic group (*p* < 0.05, Figures 4D and 4E), while the fully-allogeneic OD-PE and IVF-healthy group did not show significant differences between any other groups. Within each group, the microenvironments of CD163^+^HLA-DR^+^ myeloid cells, CD4^+^ T cells, and FoxP3^+^ T cells were compared between nulliparous and multiparous women. No significant difference was found.

### Morphologic alterations in the decidua, maternal HLA antibody status, and HLA-C/NK cell receptor haplotype combination do not explain pregnancy outcome

Given the divergent pattern of myeloid-T cell microenvironment findings in the fully-allogeneic healthy group, we further tested if outcome is related to other parameters. The extent of inflammatory lesions (deciduitis and basal villitis) in the decidua basalis was not different between groups (Table S3). Also, the frequency of maternal HLA antibody against fetal antigens did not differ between healthy and PE, although as expected the semi-allogeneic group displayed a lower maternal HLA antibody status (Figure S5). Considering previous findings on the relationship of the combination of maternal HLA-C allotype and fetal NK cell receptor (KIR) haplotype with outcome of naturally conceived pregnancies,^20^^,^^21^ we tested this in our OD cohort. Most notably, frequencies of missing self HLA-C2 in the mother and HLA-C2/KIR AA combination did not differ between groups (Table S4).

### RNA sequencing of decidua basalis reveals differentially expressed genes related to immune regulation, immune response, and oxidative stress between groups

**Figure 5:**
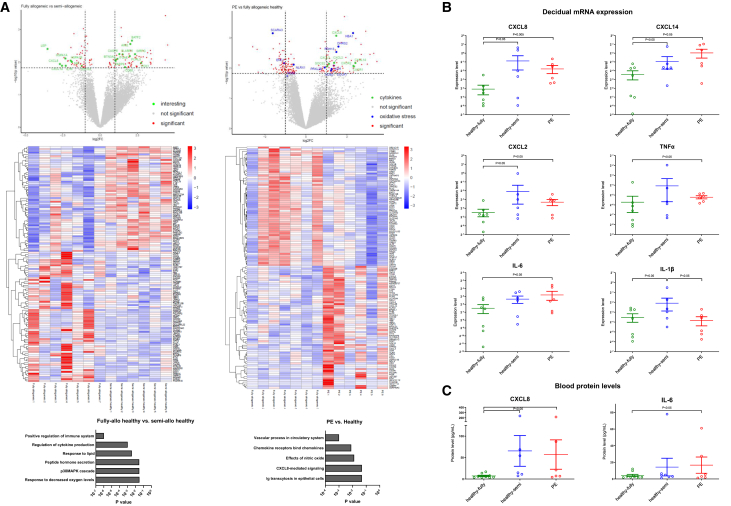
Gene expression differences between OD pregnancy groups (A) Upper: volcano plot of comparison of fully-allogeneic OD-healthy pregnancies (*n* = 7) versus semi-allogeneic OD-healthy pregnancies (*n* = 6) (left) and versus pre-eclamptic pregnancies (*n* = 6) (right). (Left) Red dots indicate DE genes (raw *p* < 0.015) and green dots indicate DE genes that are immune related (as shown in Table 3). (Right) Red dots indicate DE genes, green dots indicate DE genes that are immune-related, and blue dots indicate DE genes that are oxidative stress related (as shown in Table 4). (Middle) The corresponding heatmaps including the top 130 DEGs with raw *p* value < 0.015. (Lower) Significant pathways in each of the sets of DE genes. DE, differentially-expressed; DEG, differentially-expressed genes. (B) Gene expression levels of chemokines and cytokines in the decidua basalis of OD pregnancy groups by qPCR analysis. Signals for transcripts of interest are shown after normalization for the geometric mean signal of five reference genes. Groups were compared using Mann-Whitney U tests, and *p* values were corrected for multiple comparisons using Bonferroni correction. (C) Soluble levels of CXCL8 and IL-6 in blood plasma of the OD pregnancy groups by Luminex analysis. For each graph in (A and B), the middle horizontal line indicates the median and the whiskers indicate the upper and lower quartiles of the data. Groups were compared using Mann-Whitney U tests.

Following the differences we observed in the spatial orientation of decidual T cell- and myeloid cell subsets between pregnancy groups, we performed RNA sequencing on dissected decidua basalis from frozen placental tissue sections. First of all, we compared outcome for the fully-allogeneic healthy group (*n* = 7) to the semi-allogeneic healthy group (*n* = 6): 188 genes were differentially expressed (Figure 5A). After pathway analysis, the top biological processes included “regulation of the immune system” (*p* = 5.4 × 10^−7^), “regulation of cytokine production” (*p* = 4.2 × 10^−3^), and “response to decreased oxygen levels” (*p* = 2.7 × 10^−2^). In line with regulation of the immune system pathway, several genes (BATF2, AIM2, SIRPG, FCRL3, HSPA1, and ITGAE) have been described to be expressed in Tregs or involved in reinforcing a Treg phenotype (Table 3). Direction of their expression was in favor of the fully-allogeneic group (Table 3; Figures 5A and S6). At the same time, several genes related to cytokine/chemokine and inflammatory responses (CXCL8/IL-8, IL1B, CXCL14, MIF, and TNF) were decreased in expression in the fully-allogeneic group compared to the semi-allogeneic group (Figures 5A and S6). Hierarchical clustering of correlations between signal intensity of immune markers in the three OD pregnancy groups together indeed supported the notion of a cluster of immune regulation/Treg genes (Figure S7A, cluster 3) and a cluster of inflammation-related genes (number 2a-b). In cluster 2a, high correlations were found between CXCL8, TNF, and CXCL2, and also with HSPA1A and HSPA1B (Figure S7B) that negatively modulate Treg-suppressive activity during inflammation.^25^

**Table 3:** Immune-related genes differentially expressed between fully-allogeneic OD-healthy pregnancies and semi-allogeneic OD-healthy pregnancies

Gene	Function	AVGlog2FC^a^	*p* value
BATF2	Promotes Treg cell differentiation while inhibiting Th17 cell differentiation.^22^	2.137	0.00220
AIM2	Key regulator of Treg homeostasis by promoting their stability.	2.029	0.00279
LEP	Solely and highly expressed in EVT and SCT. Promotes Th1 cell immune responses. Increases CD4^+^CD25^−^ T cell proliferation and reduces autophagy during TCR stimulation.	−3.521	0.00395
CASP6	Plays a central role in the execution phase of cell apoptosis.	1.293	0.00569
SIRPG	Solely expressed on T cells, especially Tregs. Relevant for engagement of T cells and antigen-presenting cells. Expression related to preventing T cell hyperactivity and effector responses.^23^	2.345	0.00574
SLAMF6	Represents exhausted CD8^+^ T cells. Functions as an inhibitory receptor that controls (auto) immune responses. Promotes T cell differentiation into a Th17 phenotype.	1.514	0.00581
FCRL3	Tregs expressing FCRL3 exhibit a memory phenotype. FCRL3 positivity identifies HELIOS+ Tregs that represent an immune-suppressive cell population.^24^	1.990	0.00658
TRIM15	Plays a role in innate antiviral immunity, cell migration, and chemotaxis.	3.122	0.00727
MMP12	May be involved in tissue injury and remodeling.	−2.419	0.00731
HSPA1A	Essential for STUB1-mediated ubiquitination and degradation of FOXP3 in Tregs.^25^	−2.327	0.00779
ITGAE	Treg signature gene induced by FOXP3 in CD4^+^ T cells.^26^	1.163	0.00800
TNFSF13B	Potent B cell activator. Important role in the proliferation and differentiation of B cells.	1.103	0.00830
BTN3A3	Plays a role in T cell responses. Inhibits excessive cellular immune responses.	1.020	0.00863
PYHIN1	Major mediator of the tumor suppressor activity of IFN in breast cancer cells.	1.691	0.00866
HSPA1B	Essential for STUB1-mediated ubiquitination and degradation of FOXP3 in Tregs.^25^	−1.968	0.00906
PARP9	Plays a role in DNA damage repair and in immune responses. In macrophages, positively regulates pro-inflammatory cytokine production in response to IFNG stimulation.	1.045	0.00919
GBP5	Acts as an activator of NLRP3 inflammasome assembly.	2.194	0.00965
MIF	Pro-inflammatory cytokine. Mediator in regulating host defense by macrophages.	−1.889	0.00990
PCK1	Regulates gluconeogenesis and formation/maintenance of memory CD8^+^ T cells.	2.754	0.0106
CXCL8	Participates in the proinflammatory signaling cascade. Plays a role in inflammatory syndromes.	−2.692	0.0115
NMI	Acts as a signaling pathway regulator involved in innate immune system response.	1.024	0.0120
GCSAML	Signaling molecule associated with germinal centers.	2.114	0.0121
CXCL14	Involved in inflammatory and immunoregulatory processes.	−2.552	0.0130
IL1B	Potent pro-inflammatory cytokine. Promotes Th17 differentiation of T cells. Synergizes with IL12 to induce IFNG synthesis from Th1 cells.	−2.303	0.0130
SOCS2	Cytokine-inducible negative regulators of cytokine receptor signaling. Diseases associated with SOCS2 include placental insufficiency.	−1.225	0.0132
CFD	Catalyzes the cleavage of factor B, the alternative pathway of complement activation.	−1.551	0.0135
EFNB3	Crucial for migration and adhesion during neuronal and vascular/epithelial development.	1.923	0.0139
LRRK2	Member of the leucine-rich repeat kinase family.	0.979	0.0142
CD38	Activation marker on immune cells. Ectoenzyme that restricts TCR-involving CD4^+^ T cell responses.	1.672	0.0143

a log2 fold change of fully-allogeneic compared to semi-allogeneic.

Next, we compared outcome for the fully-allogeneic healthy group (*n* = 7) to the fully-allogeneic PE group (*n* = 6): 247 genes were differentially expressed with a *p* < 0.015 (Figure 5A). The most significant biological process pathway was represented by “vascular process in circulatory system” (*p* = 1.0 × 10^−3^). The only significant pathway by molecular function (*p* = 5 × 10^−2^) was “CXCL8/IL-8 mediated signaling.” This observation fitted with multiple inflammation-related genes (CXCL8, CXCL2, CXCL14, CXCL3, CXCR2, and CXCR1) to be expressed higher during PE (Table 4).

**Table 4:** Immune-related and oxidative stress-related genes that were differentially expressed between fully-allogeneic healthy OD pregnancies and fully-allogeneic pre-eclamptic OD pregnancies

Immune-related			
Gene	Function	AVGlog2FC^a^	*p* value
CXCL8	Participates in the proinflammatory signaling cascade. Plays a role in inflammatory syndromes.	−1.480	0.00086
CXCL2	Involved in inflammatory and immunoregulatory processes. Expressed at sites of inflammation.	−1.376	0.00671
CXCL14	Involved in inflammatory and immunoregulatory processes.	−2.412	0.00679
CXCL3	Plays a role in inflammation, and as a chemoattractant, exerts effects on endothelial cells.	−1.124	0.00723
CXCR2	Receptor (B-isoform) for CXCL-8, as well as for CXCL-1 and CXCL-3.	−2.315	0.00844
SOCS2	Negative regulator of cytokine receptor signaling. Diseases associated with SOCS2 include placental insufficiency.	−1.044	0.00866
CXCR1	Receptor (A-isoform) for CXCL-8.	−2.343	0.00883

Oxidative stress-related			
Gene	Function	AVGlog2FC^a^	*p* value
HBA1	Involved in oxygen binding.	−2.298	0.00070
SCARA3	Depletes ROS and thus plays an important role in protecting cells from oxidative stress.	1.613	0.00071
DHRS2	Can metabolize many different compounds, including xenobiotics. Reduces ROS production in cancer.	−1.586	0.00195
RDH13	May function in retinoic acid production and may also protect mitochondria against oxidative stress.	−1.486	0.00294
UGT2B7	Regulated by Nrf2 that regulates cytoprotective enzymes in response to injury and inflammation.	−2.696	0.00354
ENC1	Plays a role in the oxidative stress response as a regulator of the transcription factor Nrf2.	1.057	0.00690
NLRX1	NOD-like receptor that triggers ROS production. Prevents uncontrolled inflammation in females.	0.594	0.00769
XDH	Involved in the oxidative metabolism of purines. Contributes to the generation of ROS.	−1.358	0.00831
PRXL2B	Predicted to enable antioxidant activity and prostaglandin-F synthase activity.	−0.916	0.00917
DUOX1	Generates hydrogen peroxide and thereby plays a role in the activity of several peroxidases.	−1.255	0.0107
NOS3	Constitutive, endothelial type of NOS involved in production of nitric oxide.	−1.226	0.0121
NOS2	Produces nitric oxide that regulates vessel homeostasis and protects against hypertension.	0.900	0.0189

NOS, nucleotide-binding oligomerization domain; ROS, reactive oxygen species.

a log2 fold change of fully-allogeneic healthy OD compared to fully-allogeneic pre-eclamptic OD.

Given these findings, and in conjunction with those from the comparison with the semi-allogeneic group, we performed qPCR analyses to validate the RNA sequencing results (Figure 5B). The fully-allogeneic healthy group, indeed, showed significantly lower expression of CXCL8, CXCL14, CXCL2, and interleukin (IL)-1β than the semi-allogeneic healthy group (*p* < 0.05) and lower CXCL8, CXCL14, CXCL2, TNFα, and IL-6 (*p* < 0.05) than the fully-allogeneic PE group.

**Figure 6:**
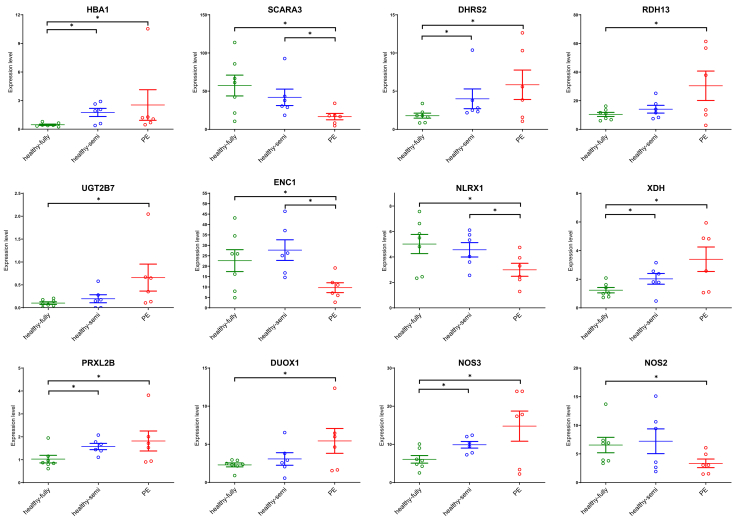
Expression of genes related to reactive oxygen species production and oxidative stress in the decidua basalis of the OD pregnancy groups The vertical axis in the graphs represents count-per-million, obtained after RNA sequencing normalization. In each graph, the middle horizontal line indicates the median and the whiskers indicate the upper and lower quartiles of the data. Groups were compared using Mann-Whitney U tests. Asterisks indicate differences with *p* < 0.015. Further details concerning the genes are shown in Table 4.

Further comparing the fully-allogeneic healthy to the fully-allogeneic PE group, the only WikiPathway identified (*p* = 1.3 × 10^−2^) was “effects of nitric oxide” (HBA1, XDH, and NOS3). This observation corresponds with our earlier findings of reactive species interactome alterations in OD pregnancies between healthy and PE.^27^ A more detailed look at the list of differentially expressed genes revealed several genes (SCARA3, DHRS2, RDH13, UGT2B7, ENC1, NLRX1, PRXL2B, DUOX1, and NOS2) related to reactive oxygen species production and oxidative stress (Table 4). These genes were differentially expressed between fully-allogeneic healthy and fully-allogeneic PE group, with the semi-allogeneic healthy often having expression levels falling in between those of the two other groups (Figure 6).

### Soluble protein markers in peripheral blood

Following gene expression differences in the decidua basalis, we assessed soluble CXCL8 protein levels in the peripheral blood. These levels showed significantly lower levels in the fully-allogeneic healthy group than in the other two groups (Figure 5C), which is in concordance with the results for the decidual tissue CXCL8 expression. Similarly, peripheral blood levels of IL-6 were significantly different between healthy and PE pregnancies. As reference, we measured levels of sFlt-1 and PlGF and the ratio between these two compounds, which were shown to indicate PE in autologous pregnancies.^28^ The OD-PE group showed significantly decreased levels of PlGF and increased sFlt1:PlGF ratio (Figure S8).

## Discussion

In the context of pregnancy, where a tolerogenic environment at the fetal-maternal interface is needed to assure a healthy course of gestation, OD pregnancies represent an interesting immunological situation. To unravel the phenotype, spatial distribution, and microenvironment of decidual immune cells in OD pregnancies, we applied *in situ* techniques on samples representing diverse pregnancy conditions. Myeloid cells emerged as the predominant cell type. Among the 12 identified myeloid clusters, those positive for CD163 and HLA-DR had higher frequencies of encountering CD4^+^ T cells in their microenvironment in the fully-allogeneic OD-healthy group compared to the semi-allogeneic group. Likewise, the former group more frequently had decidual FoxP3^+^ Tregs in the neighborhood of FoxP3^−^ (conventional) T cells. By further validation, CD163^+^HLA-DR^+^ myeloid cells and CD4^+^ T cells were more frequently in each other’s microenvironment during healthy OD pregnancy than in women with PE. Decidua of fully-allogeneic OD-healthy had a gene signature of Treg reinforcement and decreased inflammatory marker expression, whereas decidua of fully-allogeneic OD-PE typically showed increased inflammatory cytokine/chemokine expression and an imbalance in expression of oxidative stress-related genes. Differences in pregnancy outcomes could not be explained by morphologic alterations at the decidua, maternal HLA antibody status, and maternal HLA-C/fetal KIR haplotype combination.

In our study, both IMC and IF analysis did not reveal significant differences in frequencies across any decidual cell lineages or clusters. These results suggest that spatial cell orientation rather than cell frequencies is more determinative for outcome. In a previous study from our group, the percentage of FoxP3^+^ Tregs was also not different between OD pregnancies with 6–10 HLA mismatches and those with 0–5 mismatches.^18^ Our previous work using CD14 and CD163 to define decidual macrophages did not identify an association between CD14-positive staining and fetal-maternal HLA mismatches. However, we observed a correlation between the CD163^+^/CD14^+^ ratio and HLA mismatches.^19^

In alignment with a prior study from our group in autologous, naturally conceived pregnancies,^15^ the current study highlighted that in term placentas of OD pregnancies also myeloid cells represent the largest part, i.e., 65%, of the total decidual immune cell compartment. This proportion was much higher than the decidual myeloid cell percentage determined by suspension techniques (around 20%) whereby myeloid cells might have been lost during the cell isolation processes.^29^ Thus, this study suggests the prominence of myeloid cells that is comparable between OD pregnancies and naturally conceived pregnancies and again emphasizes the advantage of *in situ* analyses to study decidual immune cells, especially myeloid cells, in an unbiased manner.

Tissue myeloid cells comprise diverse subpopulations and have tissue-specific functions,^16^^,^^30^ but currently, there is a lack of universally accepted phenotypic classification besides M1/M2 polarization. We used CD163, a scavenger receptor for hemoglobin-haptoglobin complexes exclusively expressed on myeloid cells, to separate decidual myeloid cells into two major subpopulations. The CD163^+^ macrophage population has been considered to represent M2- or M2-like macrophages with immune regulatory effects.^31^ Identifying in total 12 myeloid clusters, our study further elucidated that decidual myeloid cells display a diverse spectrum of phenotypes with distinct marker expression pattern, although the precise immunological function of each cluster awaits further definition.

In the decidua tissue from OD pregnancies we detected a myeloid cluster that was also positive for NK cell markers (CD56 and CD7). The majority of this cluster consisted of single cells expressing both myeloid and NK cell markers, and this population was described in another study of naturally conceived pregnancies as well.^15^ However, in the current study, a small number of cells with double nuclei could be detected within this population, which might reflect the interaction of myeloid cells and NK cells. Cell segmentation is no longer applicable in this situation, as two nuclei that belong to different types of cells could not be separated, while pixel analysis would be more accurate to indicate cell proximity. We performed pixel analysis and found no difference in the amount of CD56/CD163 double-positive pixels between fully- and semi-allogeneic OD-healthy groups. In addition, a low amount of CD138^+^ plasma cells could be found in several healthy OD samples, which may be due to the existence of deciduitis in these samples.^32^

It has been described that most myeloid cells in the decidua are HLA-DR positive^16^ and are thereby capable of activating CD4^+^ T helper cells by presenting antigens to them. However, it is still unclear what antigens these would be. Trophoblasts, representing foreign antigens to maternal immune cells, lack most classical HLA molecules on their surface.^33^ Therefore, they escape from maternal immunological attack and might not be presented as antigens by myeloid cells. In our study, we did not find significant differences in the microenvironment among trophoblasts and maternal immune cells between pregnancies with different fetal-maternal HLA mismatches. This may be attributed to the fact that the placentas investigated were obtained at term, a stage at which trophoblasts may have limited communication with maternal immune cells, as compared to the first trimester.^15^ Previous studies reported that first-trimester trophoblasts play a critical role in the induction of decidual macrophage polarization^34^ and that soluble factors from placental tissue (composed of fetal cells) induce myeloid cells with homeostatic properties.^35^ We hypothesize that upon contact with trophoblasts early in pregnancy, maternal myeloid cells gain an HLA-DR-positive phenotype^15^ and stay positive during further gestation to assure immunological tolerance.

The exact function of the communication between CD163^+^HLA-DR^+^ myeloid cells and CD4^+^ T cells remains to be fully understood. Marker expression of individual myeloid cells by IMC particularly pointed to the presence of DC-SIGN and CD38 upon interaction with T cells. The former is involved in the regulation of T cells by antigen-presenting cells, whereas CD38 is important for macrophage function.^36^ A previous study showed that decidual CD14^+^DC-SIGN^+^ myeloid cells are closely associated to Tregs and that *ex vivo*, they induce Tregs significantly more than their DC-SIGN^−^ counterpart.^37^ The frequency of decidual FoxP3^+^ Tregs in our study was comparable to that of earlier findings.^38^^,^^39^ We observed no significant differences in the frequencies of CD163^+^HLA-DR^+^ myeloid cells and FoxP3^+^ T cells in each other’s microenvironment between pregnancies with varying levels of fetal-maternal incompatibility. However, the proximity between FoxP3^+^ T cells and other CD4^+^ (conventional) T cells was higher in fully-allogeneic healthy pregnancies than in semi-allogeneic pregnancies, which was reflected by a decidual gene signature of Treg reinforcement in the former group. Likewise, in autologous pregnancies with fetal-maternal HLA-C mismatching, CD4^+^CD25^bright^ Tregs at the decidua were not so much different in frequency, but rather in their suppressive capacity, in comparison to their counterparts in fully HLA-C-matched pregnancies^40^; interestingly, it was concluded that HLA-DR mismatching may have an additional effect on the induction of functional Tregs.^40^ Our observations suggest a potential mechanism whereby regulatory myeloid cells enhance Treg responses in affecting other T helper cells, thereby establishing an immune tolerance network in the decidua locally. Myeloid cells could also exert their influence on T helper cells or Tregs through chemokine or cytokine secretion.^41^ In an experimental setting, decidual myeloid cells were shown to increase Treg proportions when combined with total CD4^+^ T cells, which was mediated by both cell-cell contact and soluble factors.^42^ Similar findings were observed when combining CD163^+^ myeloid cells with total CD3^+^ T cells.^34^ All in all, local myeloid cell proximity observed during late pregnancy may strengthen Treg activity that is beneficial for outcome. Along this line, early pregnancy failure has been observed to display Treg insufficiency, characterized by both a disrupted FoxP3 gene signature^43^ and an impaired suppressive effect toward effector T cells.^44^

A pregnancy can lead to remodeling of maternal immunity, inducing specialized NK cells,^45^ exhausted fetal-antigen specific CD8^+^ T cells,^46^ and altered Treg transcriptomes,^47^ which potentially may affect subsequent pregnancies. Hence, we subdivided into nulliparous and multiparous women. In our cohort, the microenvironment of decidual CD163^+^HLA-DR^+^ myeloid, CD4^+^, and FoxP3^+^ T cells did not significantly differ between nulliparous and multiparous women.

**Figure 7:**
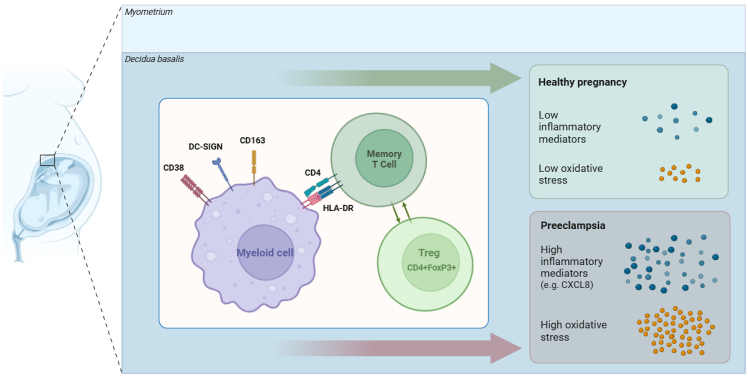
Model representing key findings of the study and further notion with regard to OD pregnancies and outcome Figure created with BioRender.com.

We hypothesize that an immune tolerance network fails to expand in the fully-allogeneic OD pregnancies with PE. A recent single-cell RNA sequencing study in term placentas during PE showed dysfunction of myeloid populations, including their ability of presenting antigens and activating T cells, at the maternal-fetal interface.^48^ Taking into account these findings, in combination with those from the current study, we hypothesize that a multiple-hit model underlies development of PE in OD pregnancies, where one factor embodies disturbed function of myeloid cells and their interaction with T cells. Another factor is the combination of oxidative stress and inflammation,^49^^,^^50^ representing the trigger for vascular and endothelial dysfunction.^51^^,^^52^ Hereby, one of the central components may be CXCL8, the expression of which we found to be elevated both at the maternal-fetal interface and in the maternal blood. The concept has been summarized in a model (Figure 7). It should be emphasized that the initial aim of our study was not to identify specific markers for PE, but the findings on RNA expression of inflammation markers and oxidative stress-related genes (Figures 5 and 6) do emphasize that the fully-allogeneic-healthy group is the odd one compared to the other two groups. Although the fully-allogeneic healthy group clearly stood out from the semi-allogeneic healthy group with respect to FoxP3+ Treg/non-Treg proximity (Figure 4) and Treg signature (Figure 5A; Table 3), this difference was not apparent in comparison to the fully-allogeneic PE group. However, it may be that the extent of exhaustion of Tregs is different, which was previously found to be increased in PE decidua.^53^ We did not find evidence of such signature in OD pregnancies with PE. Likewise, clonality of (effector) Tregs may be lower during PE compared to healthy pregnancy, as described previously,^54^ but we were not able to study this feature in our study. The fact that fetal-maternal incompatibility can be occasionally accompanied by PE might be due to the heterogeneous pathogenesis of this entity. Recent studies show that high fetal-maternal HLA antigen and epitope compatibilities in autologous donor pregnancies are associated with the presence and the severity of PE,^55^^,^^56^ suggesting that a certain amount of fetal-maternal HLA mismatches might be equally needed to maintain a healthy pregnancy.

Drawbacks of the current study may be the selective nature of inclusion of subjects. Samples were available only at term, restricting possibility of investigating possible changes during gestation. We do think that the associated materials are rather unique for research purposes. Furthermore, due to the extensiveness and complexity of data analysis and interpretation, IMC is not an ideal platform for high-throughput sample analysis. Consequently, to ensure stringent matching of clinical parameters, our initial IMC analysis was limited to a sample size of 4 versus 4. Although small, this dataset revealed a perfect group separation (U = 0, δ = 1.0, *p* = 0.029; individual data points shown in Figure 3). To directly address the statistical power limitation inherent to this small sample size, for validation we expanded the cohort to 11 versus 8 samples in multi-IF analysis. The updated Mann-Whitney U test maintains significance (U = 18, δ = −0.591, *p* = 0.033) and demonstrates substantially improved statistical power (72.8%), thereby validating the persistent biological distinction between groups. Similarly, in our multi-IF validation, we included PE samples (*n* = 8) to provide a broader pathological context. However, as our primary aim was not to delineate markers for PE but rather to underscore that the fully-allogeneic healthy OD group represents a distinct (low) immunological state, we did not perform separate sub-analyses on early-onset PE cases (*n* = 2 with gestational age <34 weeks) due to the limited sample size of subgroups. We acknowledge that exploring differences between early- and late-onset PE is a valuable pursuit but lies beyond the scope of the current study.

In conclusion, decidual myeloid cells display prominent frequencies and diverse phenotypes in OD pregnancies. When facing extreme fetal-maternal incompatibility, decidual myeloid cells turn out to have immune regulatory phenotypes (CD163^+^HLA-DR^+^) and potentially build an immune tolerance microenvironment together with T helper cells, especially Tregs, to maintain a healthy pregnancy. PE exhibits a heterogeneous pathogenesis, where dysfunctions in myeloid cell populations, reduced T cells within the microenvironment, oxidative stress, and inflammation may all play contributory roles.

### Limitations of the study

One limitation of the current study may be the selective nature of inclusion of subjects. Samples were available only at term, restricting possibility of investigating possible changes during gestation. Furthermore, as IMC is not an ideal platform for high-throughput sample analysis, initial analysis was limited to a sample size of 4 versus 4.

## Resource availability

### Lead contact

Requests for further information and resources should be directed to and will be fulfilled by the lead contact, Michael Eikmans (m.eikmans@lumc.nl).

### Materials availability

This study did not generate new unique reagents.

### Data and code availability

The raw data files of imaging mass cytometry and RNA sequencing have been deposited at https://data.mendeley.com/preview/fy8y8fnszb?a=b0176f97-a6c4-4754-814a-cc24ff2c0c1d. Any additional information required to reanalyze the data reported in this paper is available from the lead contact upon request.

## Acknowledgments

X.T. was supported by the Chinese Scholarship Council. Prof. Noel de Miranda (Dept. of Pathology, LUMC) provided technical suggestions in setting up the multilabel immunofluorescence technique.

## Author contributions

Conceptualization, X.T., L.E.E.L.O.L., M.L.P.v.d.H., and M.E.; methodology, X.T., J.K., M.E.IJ., M.L.P.v.d.H., and M.E.; validation, X.T., J.M.K., and J.D.H.A.; formal analysis: X.T., L.v.d.M., M.H.J.H., and M.E.; investigation, X.T., J.M.K., F.K., J.D.H.A., L.v.d.M., C.v.d.K., and E.v.B.; resources, L.E.E.L.O.L., M.L.P.v.d.H., and M.E.; data curation, X.T. and M.E.; software: X.T. and M.H.J.H.; writing – original draft, X.T. and M.E.; writing – review & editing: X.T., J.K., M.E.IJ., J.M.K., J.D.H.A., L.v.d.M., H.M., C.v.d.K., M.H.J.H., D.L.R., L.E.E.L.O.L., E.v.B., F.K., M.L.P.v.d.H., and M.E.; visualization, X.T. and M.E.; supervision, M.L.P.v.d.H. and M.E.; project administration, M.L.P.v.d.H. and M.E.; funding acquisition, X.T., M.L.P.v.d.H., and M.E.

## Declaration of interests

The authors declare no competing interests.

## STAR★Methods

### Key resources table

**Table:** 

REAGENT or RESOURCE	SOURCE	IDENTIFIER
**Antibodies**		

CD33	LifeSpan BioSciences	PWS44
Collagen I	Abcam	EPR7785
HLA-DR	Thermo Fisher Scientific	TAL 1B5
S100A9	Abcam	EPR3555
CD68	Cell Signaling Technology	D4B9C
CD11b	Cell Signaling Technology	D6X1N
CD4	Abcam	EPR6855
CD8α	Cell Signaling Technology	D8A8Y
CD31	Cell Signaling Technology	89C2
CD73	Cell Signaling Technology	D7F9A
CD69	Abcam	EPR21814
Granzyme B	Cell Signaling Technology	D6E9W
C1q	Abcam	C1QA/2956
Ki-67	Cell Signaling Technology	8D5
CD3	Abcam	EP449E
CD66b	BioLegend	G10F5
Flt-1	R&D Systems	AF321
HLA-G	Exbio	MEM-G2
CD39	Abcam	EPR20627
CD1c	Abcam	EPR23189-196
CD16	Cell Signaling Technology	D1N9L
CD138	BioLegend	MI15
DC-SIGN	Novus Biologicals	NBP1-77284
IDO	Cell Signaling Technology	D5J4E(TM)
CD14	Cell Signaling Technology	D7A2T
CD204	Thermo Fisher Scientific	J5HTR3
CD45RO	Cell Signaling Technology	UCHL1
CD206	Cell Signaling Technology	E6T5J
CD56	Cell Signaling Technology	E7X9M
CD103	Abcam	EPR4166(2)
CD38	Abcam	EPR4106
CD45RA	Abcam	HI100
CD15	Abcam	MC480
CD19	Cell Signaling Technology	DV4VB
CD163	Abcam	EPR14643-36
CD7	Abcam	EPR4242
CD45	Cell Signaling Technology	D9M8I
CD11c	Abcam	EP1347Y
Vimentin	Cell Signaling Technology	D21H3
Keratin	Cell Signaling Technology	C11 and AE1/AE3
α-SMA	Cell Signaling Technology	D4K9N
β-catenin	Cell Signaling Technology	D10A8
CD4	Abcam	EPR6855
FOXP3	Thermo Fisher Scientific	236A/E7
CD3	Abcam	EPR449E
CD163	Cell Signaling Technology	D6UJ1
HLA-DR	Thermo Fisher Scientific	TAL 1B5

**Chemicals, peptides, and recombinant proteins**		

Superblock solution	Thermofisher Scientific	37515
Intercalator Iridium	Fluidigm/Standard Biotools	201192A
Prolong Gold Antifade Reagent	Cell Signaling Technology	90715

**Critical commercial assays**		

AlloSeq Tx methodology	CareDx, Brisbane CA, USA	N/A
MaxPar X8 Polymere Antibody Labeling Kit	Fluidium/Standard Biotools	201300
Opal Polaris™ 7-Color Manual IHC Kit	PerkinElmer/Akoyabio	NEL861001KT
NucleoSpin® RNA extraction kit	Macherey-Nagel, Düren, Germany	740955
Bio-Plex Pro Human Cytokine Assay: IL-6 and IL-8	Bio-Rad	171BK29MR2 and 171B5008M
Luminex Discovery Assay Human Premixed Multi-Analyte Kit: sFlt1/PlGF	Biotechne/R&D Sytems	N/A
Enzyme-linked immunosorbent assay (LAT^TM^)	One Lambda, CA, USA	N/A

**Oligonucleotides**		

See Table S7 for 22 oligonucleotide sequences		

**Software and algorithms**		

HLA-EMMA software, NET framework 4.6	www.HLA-EMMA.com	N/A
InForm Cell Analysis software	Perkin-Elmer	N/A
ilastik (v1.3.3)	www.ilastik.org	N/A
CellProfiler (v2.2.0)	www.cellprofiler.org	N/A
ImaCytE	Technical University Delft, Netherlands (repository.tudelft.nl)	N/A
Cytosplore (v2.3.1)	www.cytosplore.org	N/A
g:Profiler (version e111_e.g.,58_p18_f463989d)	https://biit.cs.ut.ee/gprofiler/	N/A
Bio-Plex Manager (v6.2) software	Bio-Rad	N/A
SPSS statistics 29	IBM SPSS Software, Chicago USA	N/A
GraphPad Prism version 8 for Windows	GraphPad Software, San Diego California USA	N/A
R, v4.0.3 and v4.4.0	www.r-project.org	N/A

### Experimental model and study participant details

#### Patient selection and tissue processing

This study is a nested case-control study from the DONOR cohort^57^; a unique, prospective multicenter cohort study that includes patients with oocyte donation (OD) pregnancies in the Netherlands. For the current study, term placental samples were collected after primary C-section. For IMC staining, we gathered eight uncomplicated singleton OD pregnancies with similar demographic and clinical parameters (Table 1). For multispectral immunofluorescence staining as validation, 36 singleton pregnancies (Tables 2 and S5) were enrolled and fell into four groups based on their pregnancy conditions and fetal-maternal HLA mismatches: fully-allogeneic OD-healthy (*n* = 11), semi-allogeneic OD-healthy (*n* = 8), fully-allogeneic OD-pre-eclampsia (PE) (*n* = 8), and autologous OD (IVF)-healthy (*n* = 9). PE refers to newly onset hypertension (systolic blood pressure of ≥140 mmHg and/or diastolic blood pressure of ≥90 mmHg) and proteinuria (≥300 mg in 24 h) after 20 weeks of gestational age, or absence of proteinuria but with significant organ dysfunction.^58^ Gestational complications other than PE (maternal diabetes or gestational diabetes, preterm birth, stillbirth, intrauterine growth restriction) were excluded. Informed consent was obtained and this study was approved by the Medical Ethics Committee of the LUMC (P16.048, P08.229/228). Tissue pieces were fixed in formalin and embedded in paraffin (FFPE tissue). Serial sections (4-μm thick) were cut and dried overnight at 37°C.

### Method details

#### Assessment of fetal-maternal HLA mismatching

Maternal blood and fetal umbilical cord blood were collected for HLA typing, which was performed by the HLA typing laboratory at the LUMC. First, HLA-A, -B, -C, -DRB1, and -DQB1 typing at the split level was determined using Reverse Line Blot. All samples were preliminary chosen and separated based on the fetal-maternal HLA mismatches that were calculated manually from these results.

To verify fetal-maternal HLA mismatching at high-resolution, HLA typing was applied to all samples from the OD pregnancies using AlloSeq Tx methodology (CareDx, Brisbane CA, USA). Results of HLA-A, -B, -C, -DRB1, -DRB3/4/5, -DQB1, -DQA1, -DPB1, -DPA1 were used to calculate fetal-maternal HLA mismatches at allele level (4-digit) (Table S6). Software HLA epitope mismatch algorithm (HLA-EMMA)^59^ was applied. Allele-level results were used to determine mismatches. OD samples were divided into subgroups as described above: in the semi-allogeneic group the number of mismatches was not higher than half of the alleles per HLA locus; in the fully-allogeneic group the number of mismatches was higher than half of the alleles per HLA locus (Tables 1 and 2).

#### Assessment of HLA-C/KIR combinations

Fetal HLA-C1 and -C2 haplotypes were determined according to the four-digit HLA-C typing results, following criteria from the IMGT/HLA database (http://www.ebi.ac.uk/imgt/hla/align.html). DNA (50 ng per reaction) from maternal blood was typed by qPCR assays for KIR2DL1, KIR2DL2, KIR2DL3, KIR2DL5, KIR2DS1, KIR2DS2, KIR2DS3, KIR2DS4, KIR2DS5, and KIR3DS1 on a Via7 PCR machine (Thermofisher). Samples were distinguished in either KIR-AA or KIR-Bx haplotype following previously described criteria.^56^

#### Hematoxylin-eosin staining and morphology

After deparaffinization, the slides were stained with hematoxylin and eosin (H&E) under a routine process. Consecutive slides were used for IMC staining. Morphology of decidua basalis was determined by a placental pathologist for deciduitis and basal villitis (low or high grade).

#### Imaging mass cytometry

A total of 42 markers (Table S1) were analyzed in parallel in the tissues. All carrier-free formulations of antibodies were conjugated with heavy metals using the MaxPar X8 Polymere Antibody Labeling Kit according to the manufacturer’s protocol (Fluidium), except for CD33, CD4, Vimentin, Keratin, and α-SMA. CD33 and CD4 were stained using a secondary staining step with QDot800-labeled anti-mouse and ^145^Nd-labeled anti-rabbit secondary antibodies, respectively. Conjugation of Vimentin and Keratin antibodies to ^194^Pt and ^198^Pt, respectively, was performed using a previous protocol.^60^ Conjugation of α-SMA and ^208^Bi was performed according to an earlier protocol.^61^ All 42 antibodies and two secondary antibodies were titrated and tested for different incubation conditions to obtain the optimal signals on FFPE placenta tissue (Table S1).

#### Imaging mass cytometry staining

Eight placental slides were cut in 4-μm thick slides and stained with the IMC antibody panel at once according to a previous study.^62^ After deparaffinization and heat-induced epitope retrieval using citrate buffer the slides were incubated for 30 min with 200 μL of Superblock solution (Thermo Fisher Scientific). Next, 100 μL of CD33 and CD4 antibodies diluted in staining buffer (PBS/1%BSA) was added to the slides and incubated overnight at 4°C. The slides were washed three times for 5 min with washing buffer (PBS/1%BSA/0.05% Tween) and then incubated with 100 μL of QDot800-labeled anti-mouse (1:20) and ^145^Nd-labeled anti-rabbit (1:100) secondary antibodies for 1 h at room temperature (RT). After washing again for 3 × 5min, the slides were incubated with 100 μL of metal-labeled antibody mix for 5 h at RT. The second antibody mix was added to the slides after 3 × 5min washing and the slides were incubated overnight at 4°C in a humid chamber. After washing again for 3 × 5min, the slides were incubated for 5 min with 100 μL of Intercalator Iridium (1.25 μM, Fluidigm). The slides were washed 2 × 5min with washing buffer and 1 × 5min with demineralized water, and then dried under an airflow.

#### Imaging mass cytometry acquisition

The Hyperion was autotuned using a three-element tuning slide according to the manufacturer’s protocol (Fluidium). H&E staining on the consecutive slide was used for identify decidua area, and the Regions of Interest (ROIs) in the decidua basalis of each slide were randomly selected and ablated at 200 Hz.

#### Multispectral immunofluorescence

To verify findings from IMC, a six-marker immunofluorescence (IF) panel was applied to 36 samples using a protocol as described previously with optimization.^63^ Tissue slides were deparaffinized and blocked for endogenous peroxidase, followed by rehydrating in decreasing ethanol to demi-water and by antigen retrieval in citrate buffer. The tissues were incubated in Superblock buffer (Thermo Fisher Scientific) for 30 min at RT. For the detection of CD4, FoxP3, and CD3, tyramide signal amplification (TSA) was performed with Opal620, Opal570, and Opal520, respectively, from the Opal 7-colour manual IHC kit (PerkinElmer) according to the manufacturer’s instructions. Thereafter, the tissues were incubated with CD163 and HLA-DR antibodies overnight at RT, followed by incubation with corresponding fluorescent secondary antibodies Alx680 and Alx647, respectively, for 1h at RT. Lastly, tissues were incubated with DAPI (1 μM) for nuclear staining and mounted with Prolong Gold Antifade Reagent (Cell Signaling Technology).

For each sample, 7 to 10 ROIs with 1.4 × 1.0 mm^2^ area, covering the whole basalis area, were imaged at 20× magnification with the Vectra 3.0 Automated Quantitative Pathology Imaging System (PerkinElmer). InForm Cell Analysis software (PerkinElmer) was used for spectral separation of dyes using spectral libraries defined with single-marker immunofluorescence detection. The image of each marker in each ROI was saved separately from inForm for subsequent analysis.

#### Imaging data analysis

To analyze the decidua basalis only, villi and intervillous space were digitally removed based on the consecutive H&E staining, as well as on placental tissue structure markers in IMC and autofluorescence from trophoblasts in multispectral IF staining. Decidua basalis tissue area per sample was calculated in ImageJ by manually selecting the area per ROI. Basalis tissue area showed no significant difference between groups (data not shown). A single-cell mask was created for each ROI using the pipeline from a previous study^64^ with optimization. First, probability masks recognizing which pixels belong to which cells were created in ilastik (v1.3.3) based on DNA; EVT marker (HLA-G) (only for IMC data not for IF data); myeloid markers (CD68, CD14, and CD163 for IMC data, only CD163 for IF data) and other immune cell markers (CD45 and CD56 for IMC data, CD3 for IF data). Then these probability masks were loaded into CellProfiler (v2.2.0), with DNA as primary object and others as secondary objects to generate the final single-cell mask. To check their correctness, each cell mask was compared with the original IMC or IF image (Figures S1A and S1B).

To determine the threshold for each marker, all images for single markers were trained using semi-automated background identification in ilastik to identify signal and background, and turn into binary pixel values, as described before.^64^ Then the masks of all ROIs were loaded into ImaCytE^65^ to combine them with their corresponding binarized marker thresholds, and single-cell fcs files were exported to be further analyzed in Cytosplore (v2.3.1).^66^ For IMC analysis, after excluding cells without any marker expression, in total five tSNEs were performed (Figure S2). Firstly, a tSNE was made to separate immune cells and tissue cells. Then tSNEs were made to separate major immune cell lineages in the immune cell cluster, and separate trophoblasts in the tissue cell cluster. Another two tSNEs were composed to distinguish clusters in myeloid cells and T cells. To analyze CD163 and CD56 double-positive myeloid cells, double pixel analysis method was used as described before.^67^ For multispectral analysis, after excluding cells without any marker expression, one tSNE was used to separate and identify CD163^+^HLA-DR^+^ myeloid cells, CD3^+^CD4^+^FoxP3^-^ T cells, and CD3^+^CD4^+^FoxP3^+^ T cells (Figure S4A).

The microenvironment of decidual immune cells and trophoblasts was analyzed using the methods described by Krop et al.^15^ In brief, after loading back assigned phenotypes with cell masks in ImaCytE, the microenvironments (cell proximity distance set to 10 pixels between cells = 10 μm^2^) per phenotype were exported. The percentages of the microenvironment of each cell were computed by dividing the count of at least one co-localized cell in a 10-pixel radius by the absolute number of cells in that specific cluster. Then, the frequency of the immune cell clusters was calculated and used to subtract the expected percentage from the percentage of the observed microenvironment. The resulting percentages were compared between groups. The *Z* score was used to visualize the microenvironment percentage, showing the amount of standard deviation (SD) compared to the mean of the specific row in the heatmap. Permutation z-scores with 1.96 as cut-off were used to confirm that relevant microenvironments found do not occur at random.

To compare marker expression between clustered CD163^+^HLA-DR^+^ myeloid cells and CD4^+^ T cells and those not clustered, dM1, dM5, and CD4^+^ T cells were loaded back to ImaCytE for visualization, 5–8 cells from each of four cell type categories per sample (Figure S1C) were randomly selected. In total, 42–46 cells of each category were checked for marker expression in their raw images in MCD Viewer.

#### RNA analysis of decidua basalis

To specifically isolate decidua basalis from frozen placental sections, 40-μm thick tissue sections were placed on a glass slide to localize the basalis under a stereomicroscope and swiftly remove it from the placental villous part with a pipette tip. Then, the decidua basalis tissue was immediately placed in ML lysis buffer for RNA extraction, using the NucleoSpin RNA extraction kit (Macherey-Nagel, Düren, Germany).^68^ This procedure was carried out for fully-allogeneic healthy (*n* = 7), semi-allogeneic healthy (*n* = 6), and fully-allogeneic PE (*n* = 6). Average RNA yield, as determined on a Qubit, was 1,428 ± 464 ng. Sequencing was performed on 250 ng of RNA on the Illumina NovaSeq system by GenomeScan (Leiden, The Netherlands). A ribosomal RNA depletion step was incorporated and coverage was 30 million paired end reads (150 bp) per sample. RNAseq data were normalized between samples by count-per-million (CPM) approach, and data for single datapoints in the graphs are depicted as the number of raw reads mapped to the transcript, scaled by the number of sequencing reads in the sample. Differentially expressed (DE) genes (cut-off of raw *p* value <0.015) were determined for fully-allogeneic healthy in comparison to either semi-allogeneic healthy or to fully-allogeneic PE. Functional enrichment analysis of significant DE genes was performed using g:Profiler (version e111_e.g.,58_p18_f463989d). GeneCards was used to identify possible function and biological effects of individual genes. For validation by quantitative PCR, 4 μL of RNA (average 114 ± 37 ng) was transcribed to cDNA. PCR reactions contained 3 μL of 1:10 diluted cDNA, 0.3 μM primers, and 1:2 SybrGreen master mix, and they were performed on a ViA7 machine (Thermofisher). Real-time qPCR conditions were 95°C for 10 min, followed by 40 cycles of 95°C for 15 s and 60°C for 1 min.^68^ As normalizer for gene expression, the geometric mean signal of five reference genes (ACTB, GAPDH, HPRT1, RPL13A, HMBS) was used. Average intercorrelation among these references was r = 0.81. Primer sequences have been described in Table S7.

#### Maternal peripheral blood

Maternal blood was subjected to screening of HLA antibodies and to investigation of soluble protein markers. HLA antibodies in serum were determined by enzyme-linked immunosorbent assay (LAT, One Lambda, CA, USA) with OD readouts at 630 nm.^69^ For both HLA class I and II, the number of fetal antigens, to which antibodies were present, were calculated. Soluble compounds were measured in 50 μL of 2-fold diluted peripheral blood plasma samples on a Bio-Plex 200 machine. For IL-6 and IL-8 we used the Bio-Plex Pro Human Cytokine Assay (Bio-Rad). For the soluble form of fms related receptor tyrosine kinase 1 (sFlt-1) and placental growth factor (PlGF) we used the Luminex Discovery Assay Human Premixed Multi-Analyte Kit from Biotechne/R&D systems. Tests were performed according to the suppliers’ protocols. Bio-Plex Manager 6.2 software was used for data interpretation.

### Quantification and statistical analysis

Sample sizes and the number of replicates for each experiment are indicated in the corresponding figure legends. The way of data presentation (mostly median with variation) are also indicated in the figure legends. SPSS statistics 29 (IBM SPSS Software, Chicago USA), GraphPad Prism version 8 for Windows (GraphPad Software, San Diego California USA), and R version 4.0.3 were used for statistical analysis. After checking data distribution in histogram, all of our data are non-normally distributed. Therefore, continuous data were analyzed using Mann-Whitney U tests and Spearman’s correlation, while Fisher’s exact test was employed for nominal data. Differences in categorical data between subgroups were tested by chi-square crosstabs. Differences of mRNA levels by qPCR between groups were corrected for multiple comparisons (Bonferroni). Volcano plots and heatmaps were made using R version 4.4.0, and the Dpyler, Pheatmap, Tibble, Ggplot2, and Readxl packages. For creating the heatmap, we selected the top 130 DEG’s with *p* < 0.015, and log2 transformed these. As all samples were non-normally distributed according to the Shapiro-Wilk normality test and histograms, we chose for a Spearman correlation in the heatmaps. To improve readability, we scaled data per gene. For all tests, statistical significance was set at *p* < 0.05, unless otherwise noted.
