# Supplementary material for: Myeloid-T cell proximity is prominent in healthy pregnancies with extreme fetal-maternal HLA incompatibility
Source: iScience. 2025 Nov 21;29(1):114179. doi: 10.1016/j.isci.2025.114179 (PMC12774686; doi:10.1016/j.isci.2025.114179)
Supplement: Document S1. Figures S1–S9 and Tables S1–S7 [file mmc1.pdf]

iScience, Volume *29*

Supplemental information

Myeloid-T cell proximity is prominent in healthy pregnancies with extreme fetal-maternal HLA incompatibility

Xuezi Tian, Juliette Krop, Marieke E. Ijsselsteijn, Johanna M. Kapsenberg, Jacqueline D. H. Anholts, Lotte van der Meeren, Hailiang Mei, Michiel H.J. Huigen, Carin van der Keur, Dave L. Roelen, Lisa E.E.L.O. Lashley, Els van Beelen, Frits Koning, Marie-Louise P. van der Hoorn, and Michael Eikmans

Supplementary Figure 1

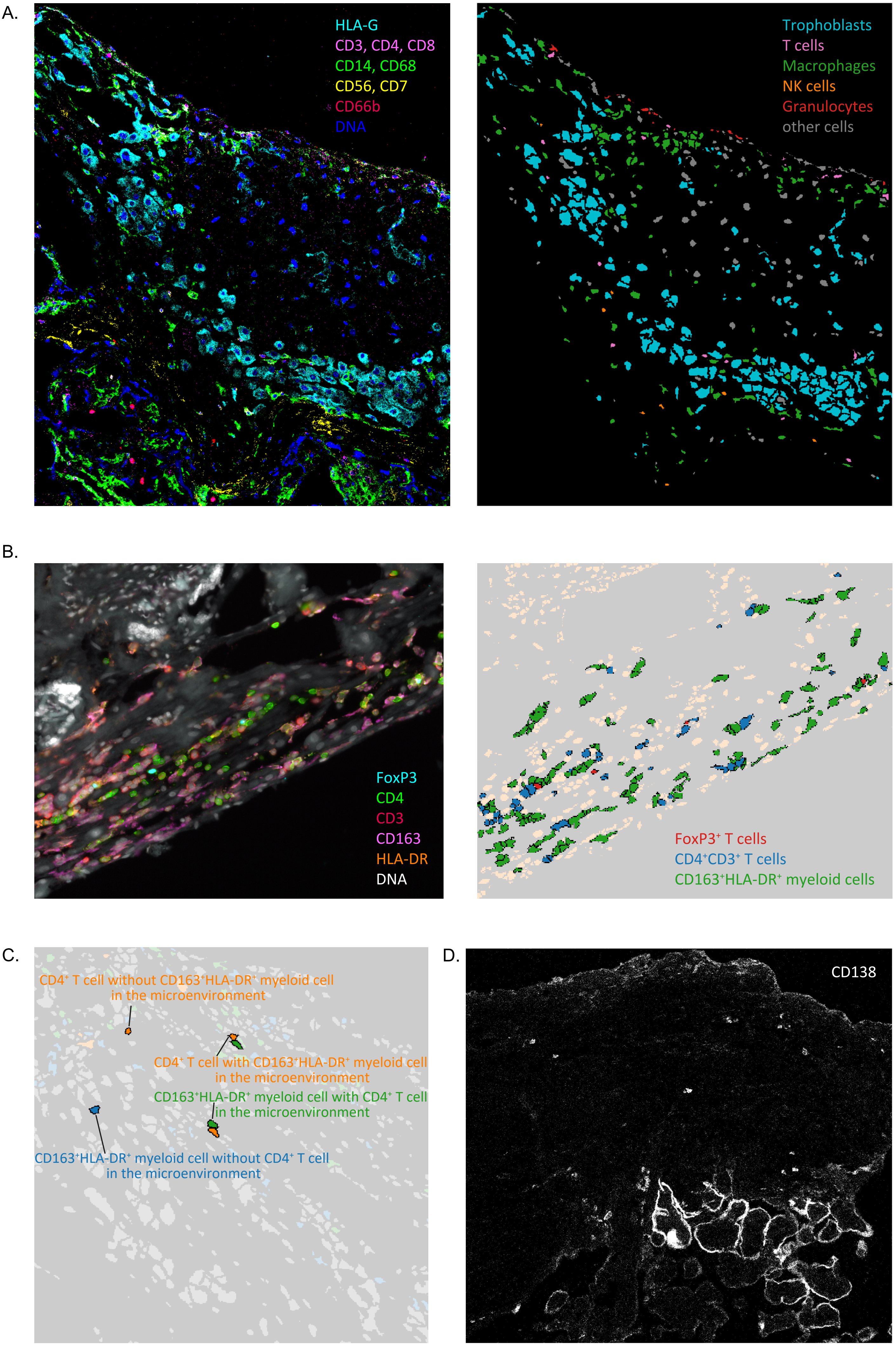

Supplementary Figure 2

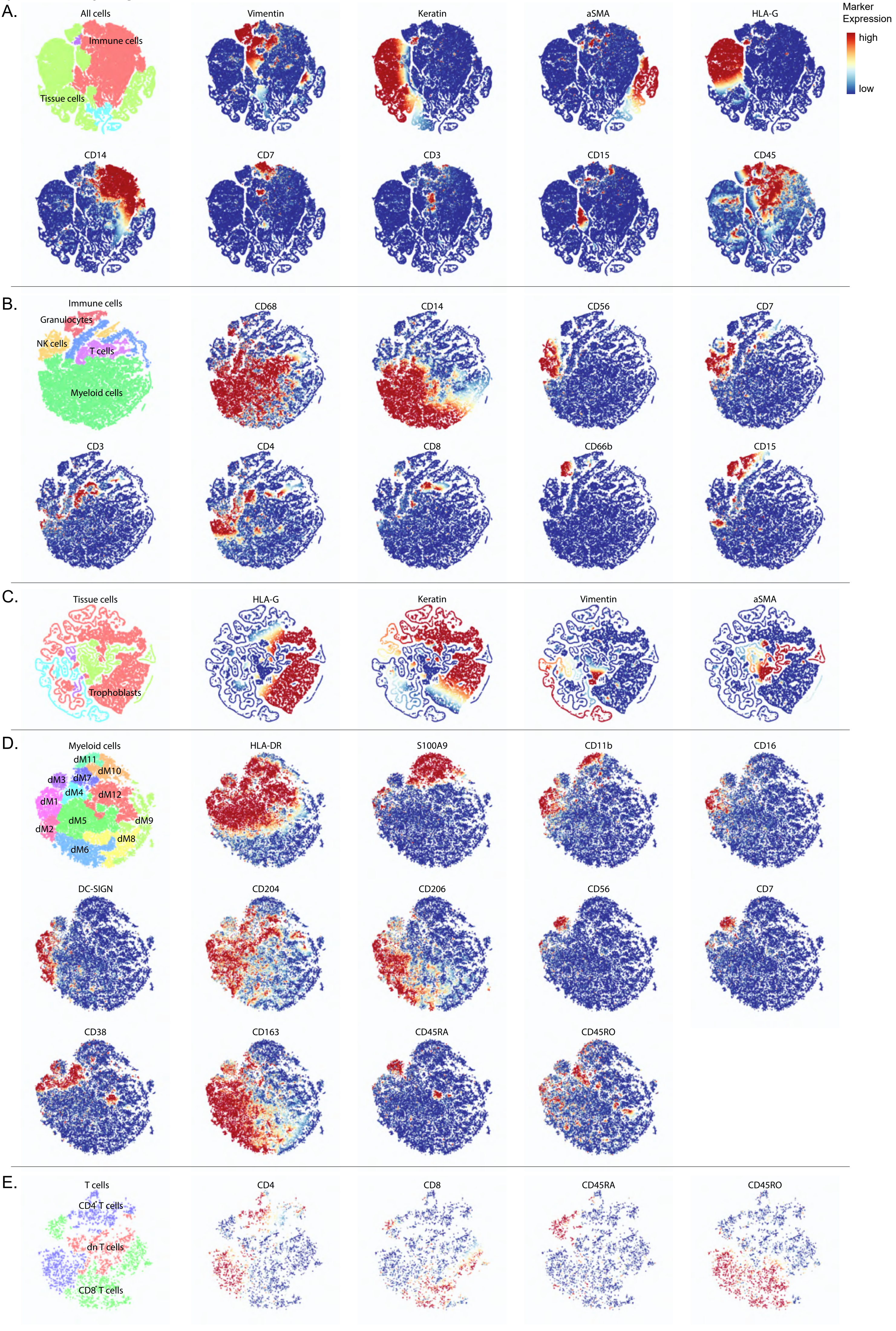

Supplementary Figure 3

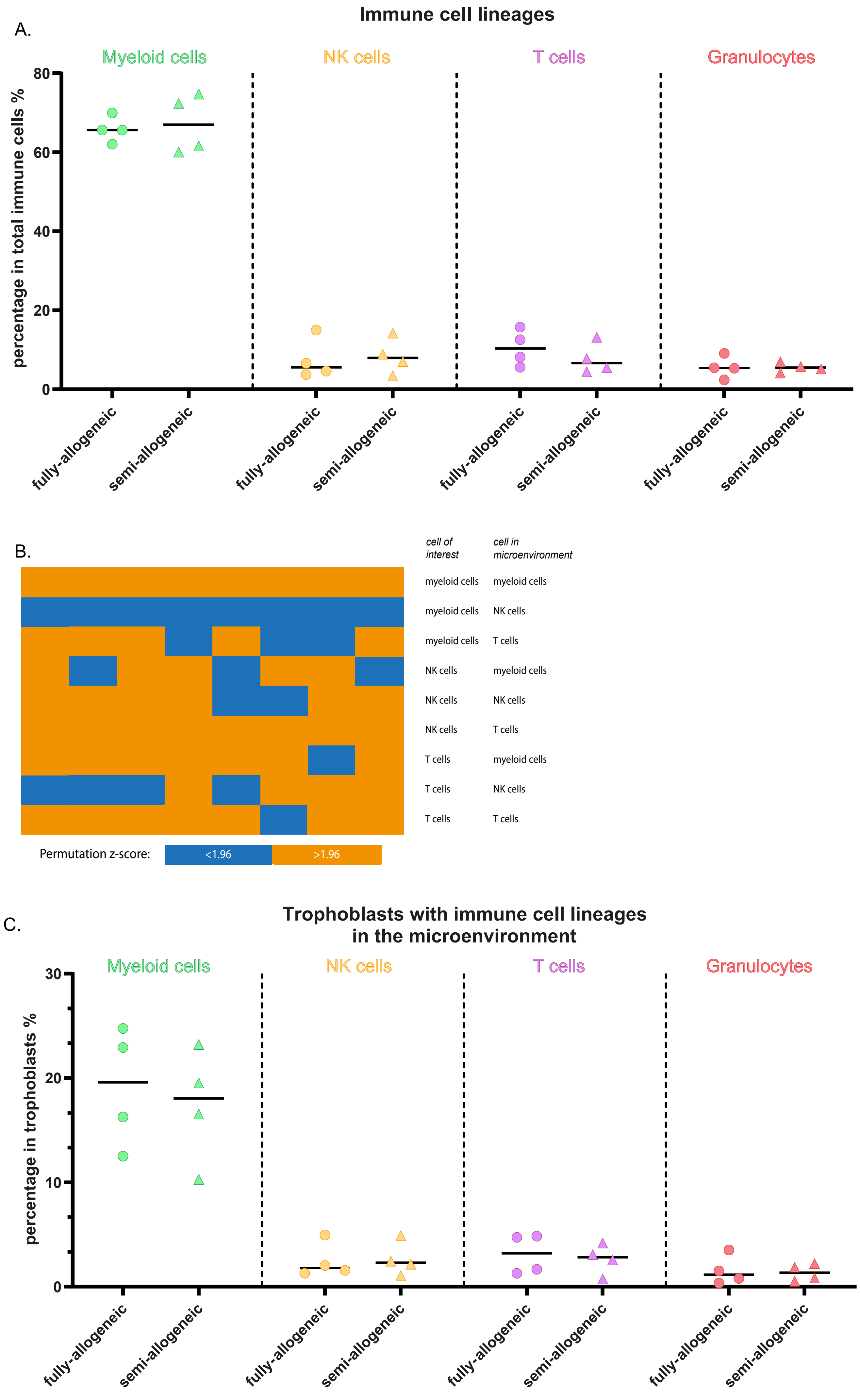

Supplementary Figure 4

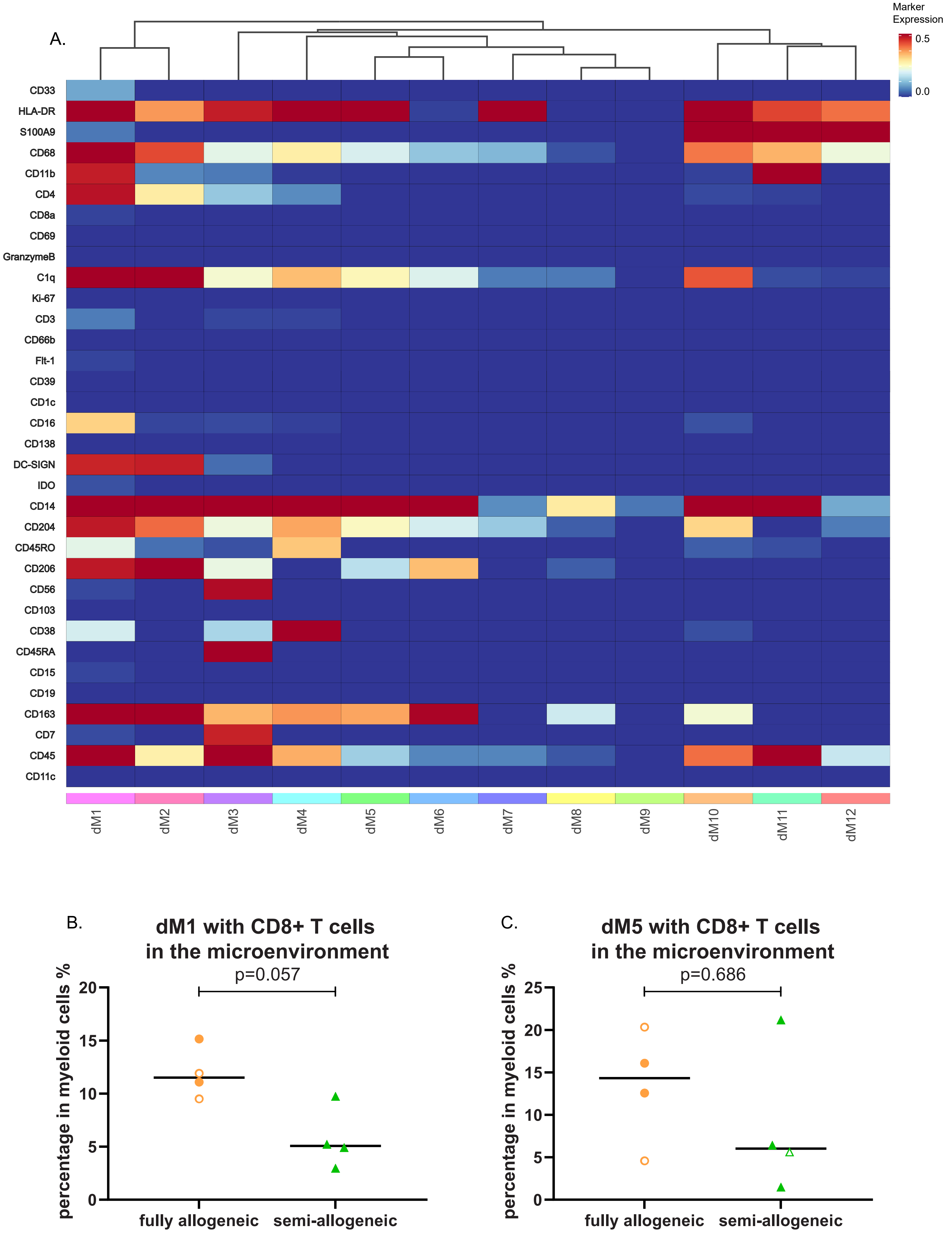

Supplementary Figure 5

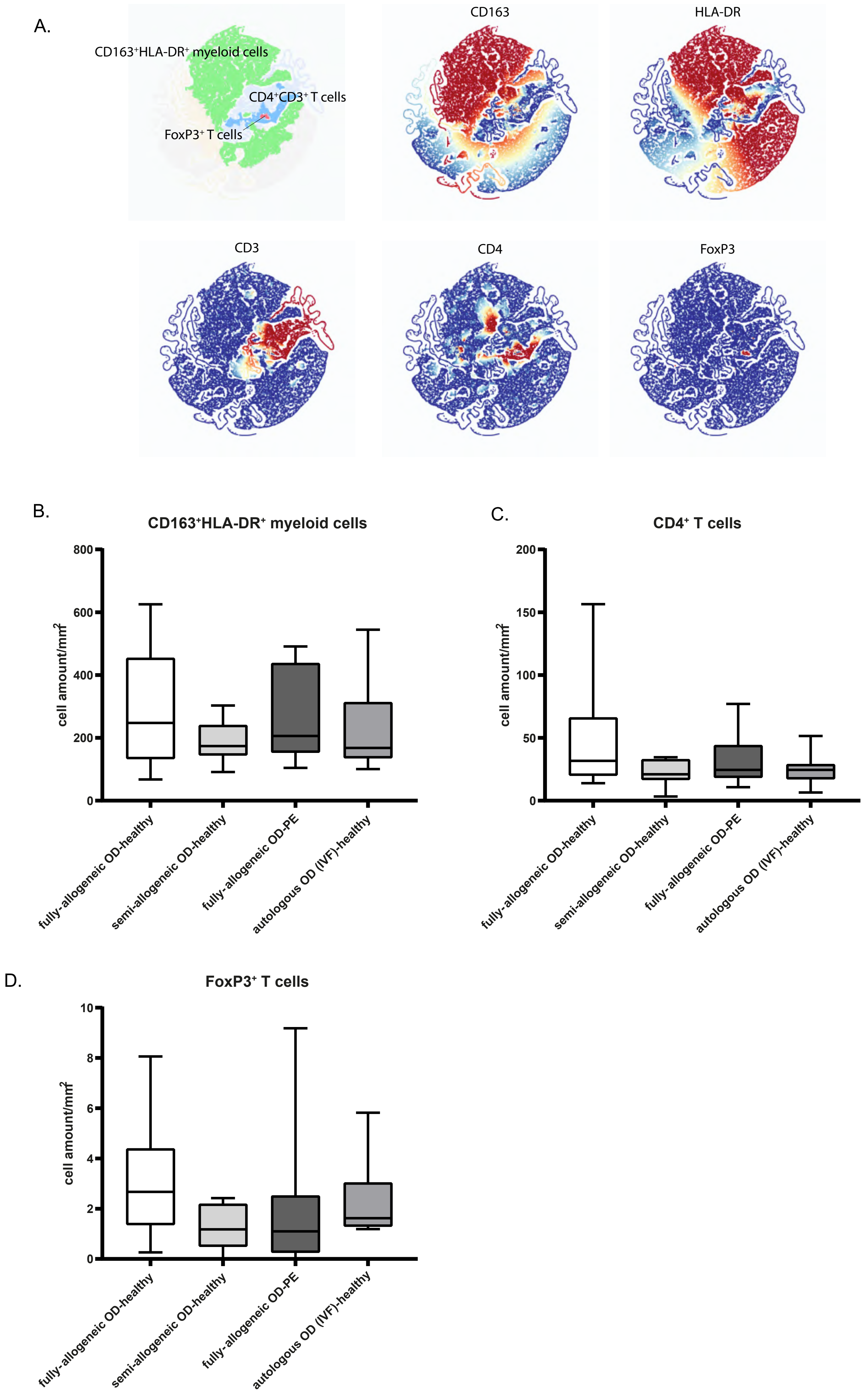

Supplementary Figure 6

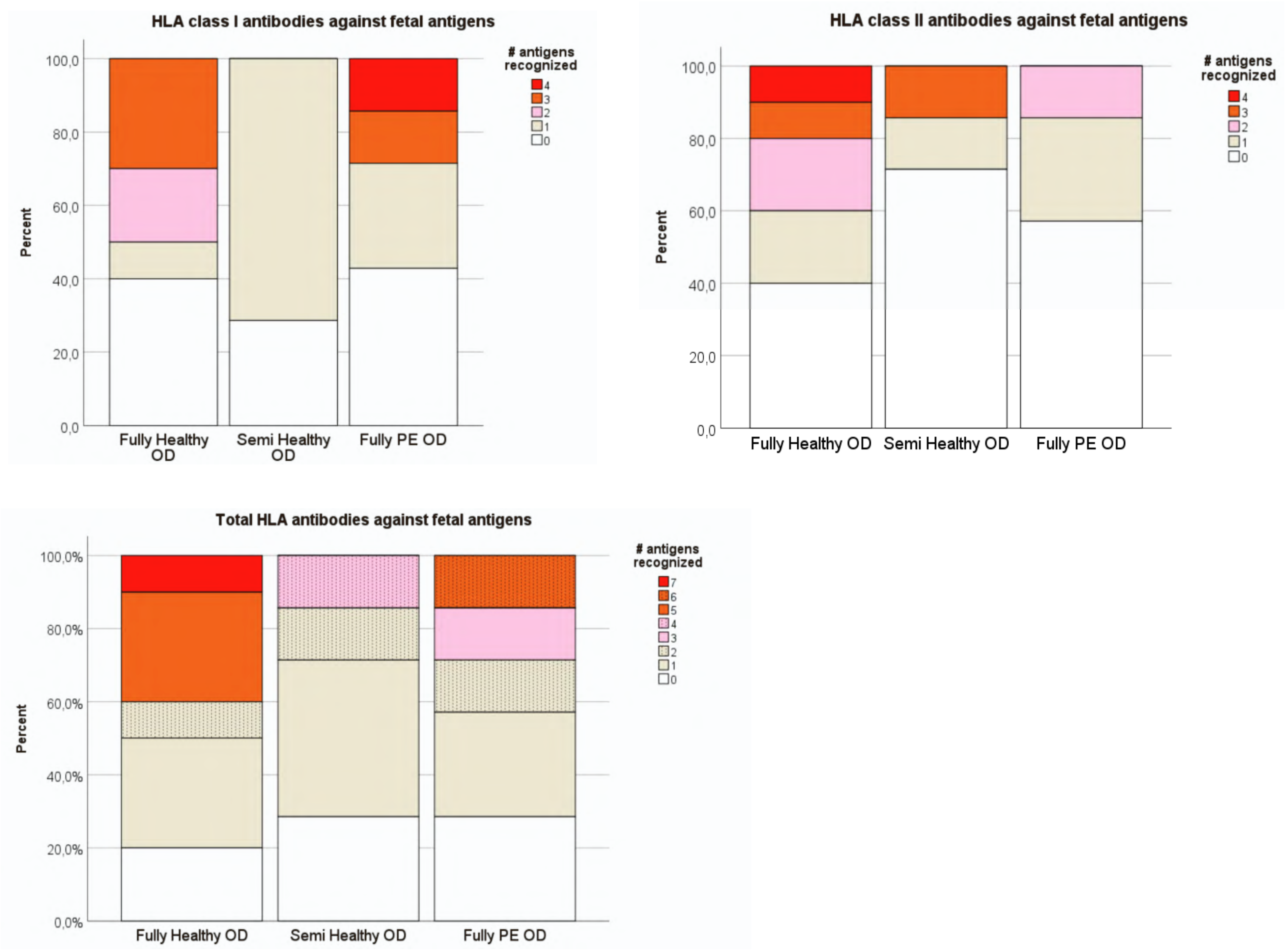

Supplementary Figure 7

Immune regulation

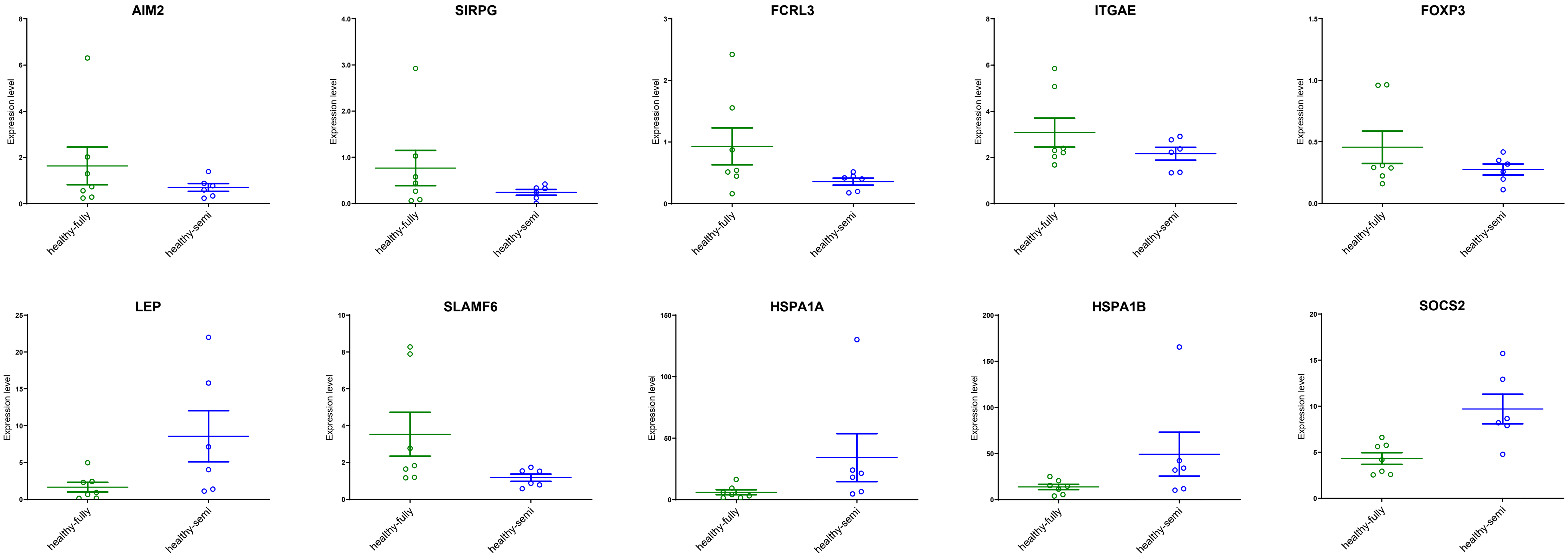

Cytokine production

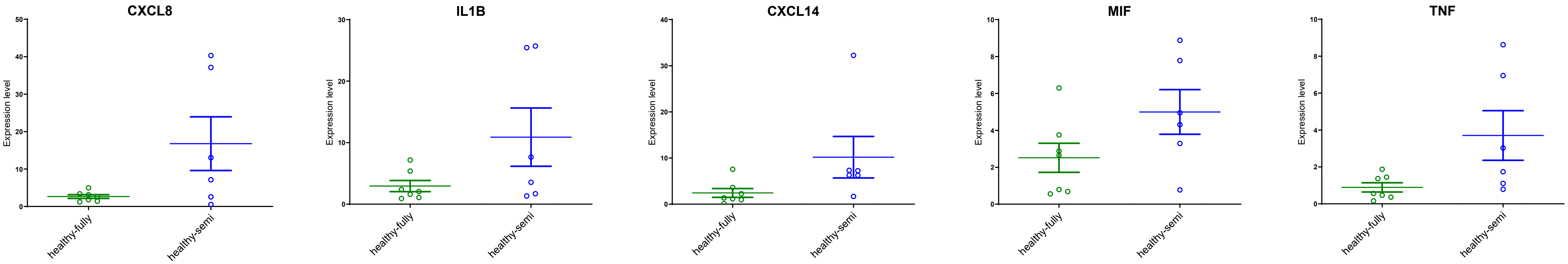

Supplementary Figure 8

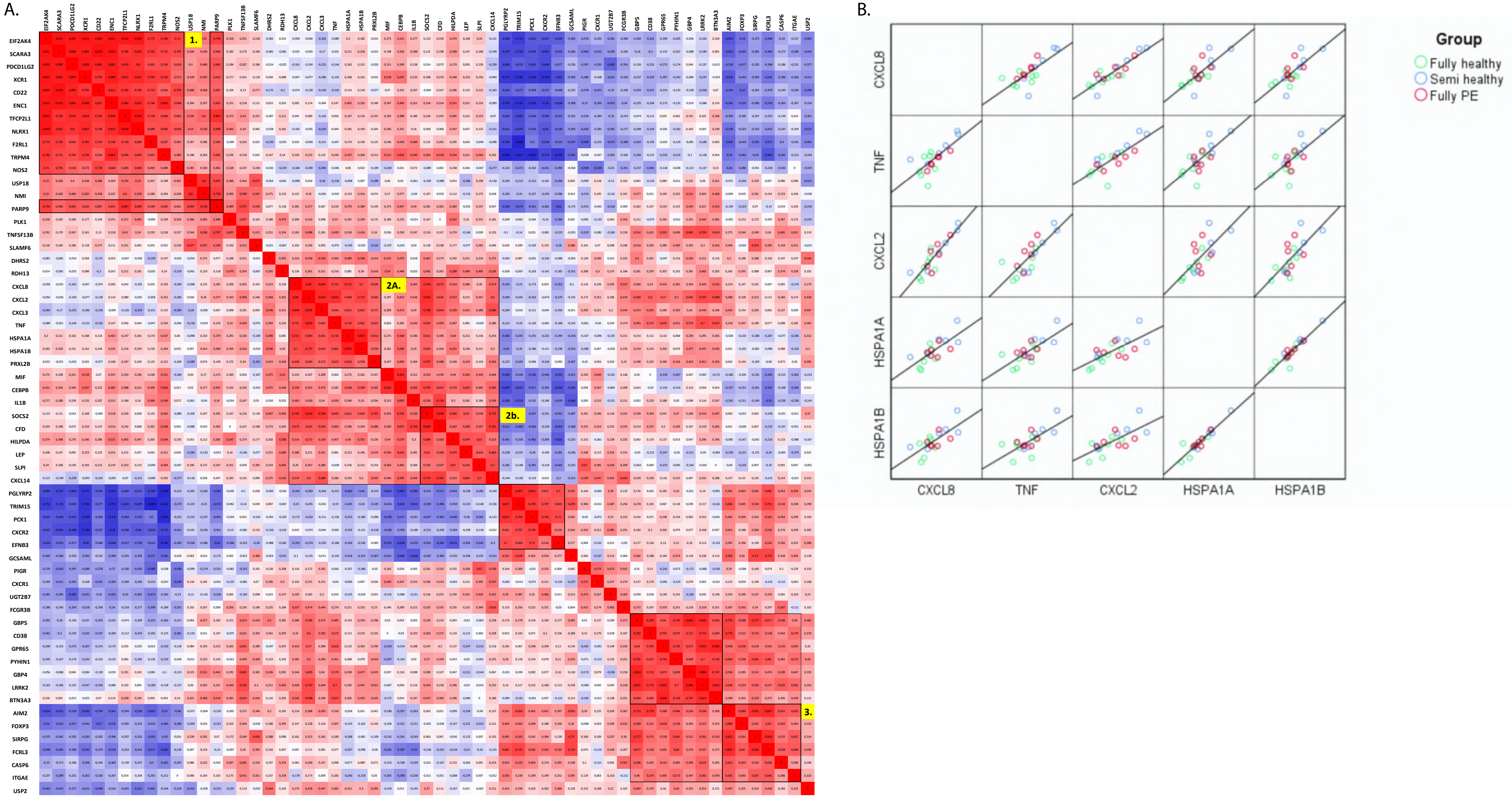

Supplementary Figure 9

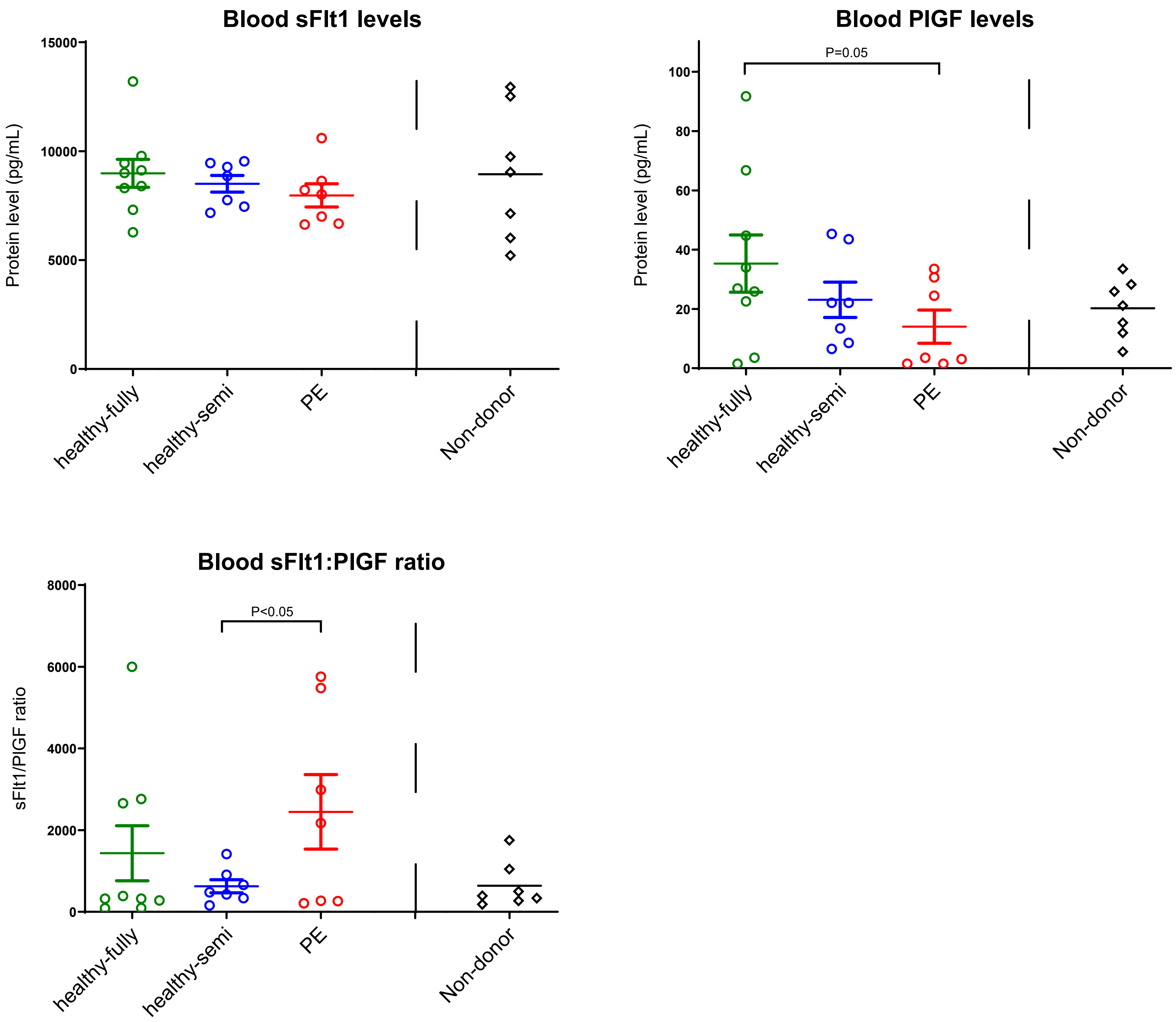

Legends to the Supplementary Figures

Supplementary Figure 1: Representative figures of major cell lineages that are detected in the decidua using imaging mass cytometry (IMC), with their corresponding cell masks. A. Villi are excluded from the cell mask, based on structure markers. **B.** Representative figures of all the markers that are detected in the decidua using multispectral immunofluorescence, with their corresponding cell masks. **C.** Representative figures of different types of individual cells that were assessed for marker expression. **D.** Representative figures of CD138 expression on the decidua, indicating the presence of plasma cells.

Supplementary Figure 2: tSNE visualization of all markers expression and clusters identified by IMC in OD-healthy samples. All clusters contain at least 100 cells. **A.** Distinction between immune cells and stromal tissue cells. **B.** Distinction between the major immune cell lineages. **C.** Distinction using structural and trophoblast markers. **D.** Distinction of different myeloid cell clusters. **E.** Distinction of T cell subclusters.

Supplementary Figure 3: A. Comparison of major immune cell lineages in decidua between fully-allogeneic (dots) and semi-allogeneic groups (triangles) in OD-healthy samples using cell percentages within total decidual immune cells. Data was shown individually, together with the median. **B.** Permutation z-scores of major immune lineage interactions in the decidua of OD-healthy samples showing that most interactions are not at random ($\geqslant 1.96$; probability of <0.05). **C.** Frequencies of the trophoblasts that are within the microenvironment of decidual immune cells in fully-allogeneic and semi-allogeneic groups. Data are shown individually, together with the median.

Supplementary Figure 4: A. Heatmap shows different marker (all immune markers in the panel) expression patterns of twelve decidual myeloid subclusters. The marker expression is binarized from 0 to 1, meaning one cell with 0.5 expression has 50% of pixels in the cell being positive for the marker. Subcluster names are displayed at the bottom of the heatmap, marker names are on the left. **B, C.** Frequencies of two myeloid cell subclusters that are within the microenvironment of CD8+T cells in fully-allogeneic and semi-allogeneic groups. Data was shown individually, together with the median. Open dots represent parous women.

Supplementary Figure 5: A. tSNE visualization of all markers expression and clusters identified by multispectral immunofluorescence in the four pregnancy groups. **B, C, D.** Cell amount per mm^2 basalis area of three cell phenotypes that can be identified using multispectral immunofluorescence on 36 samples. Data are represented as Min to Max boxplots.

Supplementary Figure 6: Maternal HLA antibody status. HLA antibodies were determined in blood serum of the mothers by Luminex analysis. The number of fetal antigens to which antibodies were present was determined for only HLA class I (left picture), for only HLA class II (middle picture), and for both classes together (right picture).

Supplementary Figure 7: Genes expression differences by RNA sequencing between fully-allogeneic and semi-allogeneic OD-healthy pregnancies. In each graph, the middle horizonal line indicates the median and the whiskers indicate the upper and lower quartiles of the data. Details concerning genes and statistical comparisons between groups are further detailed in Table 3.

Supplementary Figure 8: Correlations between genes in decidua basalis of all OD pregnancy samples following RNA sequencing. A. Hierarchical clustering of correlations between signal intensity of immune-related- and oxidative-stress-related markers. Boxes numbered with 1, 2a, 2b,

and 3 indicate sets of markers that show the highest correlation (deep red colored) with each other, whereby cluster 2 is enriched for cytokines and chemokines and cluster 3 by Treg-related markers. **B.** Levels of CXCL2, CXCL8, TNF, HSPA1A, and HSPA1B correlated significantly and positively with each other.

Supplementary Figure 9: Peripheral blood levels of sFlt-1, PlGF, and their ratio in different groups. In each graph, the middle horizonal line indicates the median and the whiskers indicate the upper and lower quartiles of the data. Comparison with significant differences are showed with p value.

Table S1. Imaging mass cytometry antibody panel

	Target	Clone	Metal	Incubation time	Temperature	Dilution	Supplier
1	CD33	PWS44	^{112}Cd/^{114}Cd	Indirect	4°C	20	LifeSpan BioSciences
2	Collagen I	EPR7785	^{115}In	5 hours	RT	50	Abcam
3	HLA-DR	TAL 1B5	^{141}Pr	5 hours	RT	100	Thermo Fisher Scientific
4	S100A9	EPR3555	^{142}Nd	Overnight	4°C	200	Abcam
5	CD68	D4B9C	^{143}Nd	Overnight	4°C	100	Cell Signaling Technology
6	CD11b	D6X1N	^{144}Nd	5 hours	RT	100	Cell Signaling Technology
7	CD4	EPR6855	^{145}Nd	Indirect	4°C	100	Abcam
8	CD8α	D8A8Y	^{146}Nd	Overnight	4°C	50	Cell Signaling Technology
9	CD31	89C2	^{147}Sm	5 hours	RT	100	Cell Signaling Technology
10	CD73	D7F9A	^{148}Nd	5 hours	RT	100	Cell Signaling Technology
11	CD69	EPR21814	^{149}Sm	Overnight	4°C	100	Abcam
12	Granzyme B	D6E9W	^{150}Nd	5 hours	RT	100	Cell Signaling Technology
13	C1q	C1QA/2956	^{151}Eu	Overnight	4°C	100	Abcam
14	Ki-67	8D5	^{152}Sm	Overnight	4°C	100	Cell Signaling Technology
15	CD3	EP449E	^{153}Eu	5 hours	RT	50	Abcam
16	CD66b	G10F5	^{154}Sm	5 hours	RT	100	BioLegend
17	Flt-1	AF321	^{155}Gd	Overnight	4°C	200	R&D Systems
18	HLA-G	MEM-G2	^{156}Gd	5 hours	RT	100	Exbio
19	CD39	EPR20627	^{157}Gd	5 hours	RT	50	Abcam
20	CD1c	EPR23189-196	^{158}Gd	Overnight	4°C	50	Abcam
21	CD16	D1N9L	^{159}Tb	Overnight	4°C	50	Cell Signaling Technology
22	CD138	MI15	^{160}Gd	Overnight	4°C	100	BioLegend
23	DC-SIGN	NBP1-77284	^{161}Dy	Overnight	4°C	50	Novus Biologicals
24	IDO	D5J4E(TM)	^{162}Dy	Overnight	4°C	100	Cell Signaling Technology
25	CD14	D7A2T	^{163}Dy	5 hours	RT	100	Cell Signaling Technology
26	CD204	J5HTR3	^{164}Dy	5 hours	RT	50	Thermo Fisher Scientific
27	CD45RO	UCHL1	^{165}Ho	Overnight	4°C	100	Cell Signaling Technology
28	CD206	E6T5J	^{166}Er	5 hours	RT	100	Cell Signaling Technology

29	CD56	E7X9M	^{167}Er	5 hours	RT	100	Cell Signaling Technology
30	CD103	EPR4166(2)	^{168}Er	Overnight	4°C	50	Abcam
31	CD38	EPR4106	^{169}Tm	Overnight	4°C	50	Abcam
32	CD45RA	HI100	^{170}Er	5 hours	RT	100	Abcam
33	CD15	MC480	^{171}Yb	Overnight	4°C	100	Abcam
34	CD19	DV4VB	^{172}Yb	Overnight	4°C	100	Cell Signaling Technology
35	CD163	EPR14643-36	^{173}Yb	5 hours	RT	100	Abcam
36	CD7	EPR4242	^{174}Yb	Overnight	4°C	100	Abcam
37	CD45	D9M8I	^{175}Lu	Overnight	4°C	100	Cell Signaling Technology
38	CD11c	EP1347Y	^{176}Yb	5 hours	RT	50	Abcam
39	Vimentin	D21H3	^{194}Pt	Overnight	4°C	50	Cell Signaling Technology
40	Keratin	C11 and AE1/AE3	^{198}Pt	Overnight	4°C	50	Cell Signaling Technology
41	α-SMA	D4K9N	^{209}Bi	5 hours	RT	100	Cell Signaling Technology
42	β-catenin	D10A8	^{89}Y	Overnight	4°C	100	Cell Signaling Technology

RT: room temperature.

Table S2. Multispectral immunofluorescence antibody panel

	Target	Clone	Species	Isotype	Detection	Fluorochrome	Dilution	Supplier
1	CD4	EPR6855	Rabbit	IgG	Opal	Opal620	1000	Abcam
2	FOXP3	236A/E7	Mouse	IgG1	Opal	Opal570	250	Thermo Fisher Scientific
3	CD3	EPR449E	Rabbit	IgG	Opal	Opal520	500	Abcam
4	CD163	D6UJ1	Rabbit	IgG	indirect	Alx680	50	Cell Signaling Technology
5	HLA-DR	TAL 1B5	Mouse	IgG1	indirect	Alx647	100	Thermo Fisher Scientific
6	DAPI							

Table S3. Pathologic lesions in decidua basalis [1]

	Normal	Deciduitis	Low grade basal villitis	High grade basal villitis
Fully-allo healthy	45.5 %	27.8 %	27.8 %	0 %
Semi-allo healthy	62.5 %	37.5 %	25 %	12.5 %
Fully-allo PE	75.0 %	25.0 %	0 %	0 %
Non-donor healthy	88.9 %	0 %	0 %	11.1 %

[1] Values indicate the frequency by which the condition or lesion was observed in the total cases per subgroup.

Table S4. HLA-C allotype / KIR haplotype combinations

	Mother: no C1/C2 missing self	Mother: C1 missing self	Mother: C2 missing self
Fully-allo healthy	8 (72.7%)	0	3 (27.3%)
Semi-allo healthy	7 (77.8%)	0	2 (22.2%)
Fully-allo PE	4 (57.1%)	1 (14.3%)	2 (28.6%)

	Fetus equal/less C2 than mother	Fetus more C2 than mother
Fully-allo healthy	5 (45.5%)	6 (54.5%)
Semi-allo healthy	7 (77.8%)	2 (22.2%)
Fully-allo PE	5 (71.4%)	2 (28.6%)

	HLA-C2 / KIR AA combination	Other combination
Fully-allo healthy	2 (18.2%)	9 (81.8%)
Semi-allo healthy	0	9 (100%)
Fully-allo PE	0	7 (100%)

Table S5. Tests performed on samples

Group	Sample number	IMC	IF	RNA
Fully allogeneic OD-healthy	Sample1	√	√	√
	Sample2	√	√	√
	Sample3	√	√	√
	Sample4	√	√	√
	Sample5		√	√
	Sample6		√	√
	Sample7		√	
	Sample8		√	
	Sample9		√	
	Sample10		√	
	Sample11		√	
Semi-allogeneic OD-healthy	Sample12	√	√	√
	Sample13	√	√	√
	Sample14	√	√	√
	Sample15	√	√	√
	Sample16		√	√
	Sample17		√	√
	Sample18		√	
	Sample19		√	
Fully allogeneic OD-PE	Sample20		√	√
	Sample21		√	√
	Sample22		√	√
	Sample23		√	√
	Sample24		√	√
	Sample25		√	√
	Sample26		√	
	Sample27		√	
IVF-healthy	Sample28		√	√
	Sample29		√	√
	Sample30		√	√
	Sample31		√	√

	Sample32		√	√
	Sample33		√	√
	Sample34		√	
	Sample35		√	
	Sample36		√	

Table S6. High-resolution HLA typing of OD samples

		HLA-A		HLA-B		HLA-C		HLA-DRB1		HLA-DRB3/4/5		HLA-DQB1		HLA-DQA1		HLA-DPB1		HLA-DPA1	
Sample1	Maternal	A*02:01	A*03:01	B*35:01	B*57:01	C*04:01	C*07:01	DRB1*04:02	DRB1*07:01	DRB4*01:03	DRB4*01:03	DQB1*03:02	DQB1*03:03	DQA1*02:01	DQA1*03:01	DPB1*02:01		DPA1*01:03	
	Fetal	A*01:01	A*26:01	B*37:01	B*51:01	C*02:02	C*06:02	DRB1*03:01	DRB1*04:01	DRB3*02:02	DRB4*01:03N	DQB1*02:01	DQB1*03:01	DQA1*03:03	DQA1*05:01	DPB1*03:01	DPB1*04:01	DPA1*01:03	
Sample2	Maternal	A*03:01		B*07:02	B*35:01	C*07:02		DRB1*14:54	DRB1*15:01	DRB3*02:02	DRB5*01:01	DQB1*05:03	DQB1*06:02	DQA1*01:02	DQA1*01:04	DPB1*02:01	DPB1*04:01	DPA1*01:03	
	Fetal	A*01:01	A*02:01	B*08:01	B*27:05	C*02:02	C*07:01	DRB1*03:01	DRB1*04:01	DRB3*01:01	DRB4*01:03	DQB1*02:01	DQB1*03:01	DQA1*03:03	DQA1*05:01	DPB1*04:01	DPB1*09:01	DPA1*01:03	DPA1*02:01
Sample3	Maternal	A*02:01	A*26:01	B*38:01	B*44:02	C*05:01	C*12:03	DRB1*13:01		DRB3*01:01		DQB1*06:03		DQA1*01:03		DPB1*04:01	DPB1*14:01	DPA1*01:03	DPA1*02:01
	Fetal	A*02:01	A*23:01	B*13:02	B*44:03	C*04:01	C*06:02	DRB1*07:01	DRB1*07:01	DRB4*01:01	DRB4*01:03	DQB1*02:02		DQA1*02:01		DPB1*04:01	DPB1*20:01	DPA1*01:03	
Sample4	Maternal	A*02:01		B*07:02	B*13:02	C*06:02	C*07:02	DRB1*07:01	DRB1*15:01	DRB4*01:03	DRB5*01:01	DQB1*02:02	DQB1*06:02	DQA1*01:02	DQA1*02:01	DPB1*04:01		DPA1*01:03	
	Fetal	A*02:01	A*24:02	B*27:02	B*38:01	C*02:02	C*12:03	DRB1*04:02	DRB1*16:01	DRB4*01:03	DRB5*02:02	DQB1*03:02	DQB1*05:02	DQA1*01:02	DQA1*03:01	DPB1*04:01	DPB1*04:02	DPA1*01:03	
Sample5	Maternal	A*01:01	A*02:01	B*08:01	B*41:01	C*07:01	C*17:01	DRB1*03:01	DRB1*07:01	DRB3*01:01	DRB4*01:01	DQB1*02:01	DQB1*03:03	DQA1*02:01	DQA1*05:01	DPB1*03:01	DPB1*04:02	DPA1*01:03	
	Fetal	A*31:01		B*27:05	B*35:01	C*02:02	C*04:01	DRB1*01:01	DRB1*04:01	DRB4*01:03		DQB1*03:02	DQB1*05:01	DQA1*01:01	DQA1*03:03	DPB1*350:01	DPB1*914:01	DPA1*01:03	
Sample6	Maternal	A*02:01		B*44:03	B*51:07	C*07:06	C*14:02	DRB1*07:01	DRB1*13:01	DRB3*01:01	DRB4*01:03	DQB1*02:02	DQB1*06:03	DQA1*01:03	DQA1*02:01	DPB1*04:01	DPB1*19:01	DPA1*01:03	DPA1*02:07
	Fetal	A*03	A*24	B*39:06	B*44:02	C*05:01	C*07:02	DRB1*08:01	DRB1*11:04	DRB3*02:02		DQB1*03:01	DQB1*04:02	DQA1*04:01	DQA1*05:05	DPB1*04:01	DPB1*04:02	DPA1*01:03	
Sample7	Maternal	A*01:01	A*31:01	B*08:01	B*40:01	C*03:04	C*07:01	DRB1*03:01	DRB1*07:01	DRB3*01:01	DRB4*01:01	DQB1*02:01	DQB1*02:02	DQA1*02:01	DQA1*05:01	DPB1*02:01	DPB1*04:02	DPA1*01:03	
	Fetal	A*01:01		B*38:01	B*40:01	C*03:04	C*12:03	DRB1*04:03	DRB1*13:02	DRB3*03:01	DRB4*01:03	DQB1*03:02	DQB1*06:04	DQA1*01:02	DQA1*03:01	DPB1*04:02	DPB1*14:01	DPA1*01:03	DPA1*02:01
Sample8	Maternal	A*02:01	A*03:01	B*07:02	B*44:02	C*05:01	C*07:02	DRB1*13:01	DRB1*15:01	DRB3*01:01	DRB5*01:01	DQB1*06:02	DQB1*06:03	DQA1*01:02	DQA1*01:03	DPB1*03:01	DPB1*04:01	DPA1*01:03	
	Fetal	A*24:02	A*25:01	B*07:02	B*51:01	C*02:02	C*07:02	DRB1*01:01	DRB1*04:04	DRB4*01:03		DQB1*03:02	DQB1*05:01	DQA1*01:01	DQA1*03:01	DPB1*02:01	DPB1*04:02	DPA1*01:03	
Sample9	Maternal	A*01:01	A*68:01	B*08:01	B*35:01	C*04:01	C*07:01	DRB1*01:01	DRB1*03:01	DRB3*01:01		DQB1*02:01	DQB1*05:01	DQA1*01:01	DQA1*05:01	DPB1*03:01	DPB1*04:01	DPA1*01:03	
	Fetal	A*02:01	A*03:01	B*15:01	B*18:01	C*03:03	C*07:01	DRB1*10:01	DRB1*13:01	DRB3*02:02		DQB1*05:01	DQB1*06:03	DQA1*01:03	DQA1*01:05	DPB1*04:01	DPB1*19:01	DPA1*01:03	DPA1*02:07
Sample10	Maternal	A*02:01	A*03:01	B*44:02	B*51:01	C*07:02	C*07:04	DRB1*07:01	DRB1*15:01	DRB4*01:01	DRB5*01:01	DQB1*02:02	DQB1*06:02	DQA1*01:02	DQA1*02:01	DPB1*02:01	DPB1*04:01	DPA1*01:03	
	Fetal	A*02:01	A*24:03	B*13:02	B*35:01	C*04:01	C*06:02	DRB1*01:01	DRB1*04:02	DRB4*01:03		DQB1*03:02	DQB1*05:01	DQA1*01:01	DQA1*03:01	DPB1*04:01	DPB1*20:01	DPA1*01:03	
Sample11	Maternal	A*03:01	A*24:02	B*07:02	B*52:01	C*07:02	C*12:02	DRB1*03:01	DRB1*13:01	DRB3*01:01	DRB3*02:02	DQB1*02:01	DQB1*06:03	DQA1*01:03	DQA1*05:01	DPB1*02:01	DPB1*14:01	DPA1*01:03	DPA1*02:01
	Fetal	A*02:01	A*03:01	B*08:01	B*49:01	C*07:01	C*07:01	DRB1*03:01	DRB1*04:05	DRB3*01:01	DRB4*01:03	DQB1*02:01	DQB1*03:01	DQA1*05:01	DQA1*05:05	DPB1*04:01	DPB1*20:01	DPA1*01:03	

Sample12	Maternal	A*01:01	A*03:01	B*08:01	B*51:01	C*04:01	C*07:01	DRB1*03:01	DRB1*11:01	DRB3*01:01	DRB3*02:02	DQB1*02:01	DQB1*03:01	DQA1*05:01	DQA1*05:05	DPB1*02:01	DPB1*05:01	DPA1*01:03	DPA1*02:01
	Fetal	A*01:01	A*03:01	B*15:01	B*44:02	C*05:01	C*07:02	DRB1*03:01	DRB1*11:01	DRB3*01:01	DRB3*02:02	DQB1*02:01	DQB1*03:01	DQA1*05:01	DQA1*05:05	DPB1*01:01	DPB1*02:01	DPA1*01:03	DPA1*02:01
Sample13	Maternal	A*02:01	A*24:02	B*08:01	B*38:01	C*07:01	C*12:03	DRB1*01:01	DRB1*03:01	DRB3*01:01		DQB1*02:01	DQB1*05:01	DQA1*01:01	DQA1*05:01	DPB1*02:01	DPB1*04:01	DPA1*01:03	
	Fetal	A*02:01	A*03:01	B*07:02	B*13:02	C*06:02	C*07:01	DRB1*01:01	DRB1*07:01	DRB4*01:03		DQB1*02:02	DQB1*05:01	DQA1*01:01	DQA1*02:01	DPB1*03:01	DPB1*04:01	DPA1*01:03	
Sample14	Maternal	A*02:01	A*03:01	B*07:02	B*51:01	C*07:02	C*14:02	DRB1*08:01	DRB1*15:01	DRB5*01:01		DQB1*04:02	DQB1*06:02	DQA1*01:02	DQA1*04:02	DPB1*03:01	DPB1*15:01	DPA1*01:03	
	Fetal	A*01:01	A*03:01	B*07:02	B*08:01	C*07:01	C*07:02	DRB1*04:04	DRB1*15:01	DRB4*01:03	DRB5*01:01	DQB1*03:02	DQB1*06:02	DQA1*01:02	DQA1*03:01	DPB1*04:01	DPB1*06:01	DPA1*01:03	
Sample15	Maternal	A*03:01	A*29:02	B*07:02	B*44:03	C*07:02	C*16:01	DRB1*07:01	DRB1*15:01	DRB4*01:01	DRB5*01:01	DQB1*02:02	DQB1*06:02	DQA1*01:02	DQA1*02:01	DPB1*04:01	DPB1*11:01	DPA1*01:03	DPA1*02:01
	Fetal	A*03:01	A*24:02	B*07:02	B*40:01	C*03:04	C*07:02	DRB1*13:02	DRB1*15:01	DRB3*03:01	DRB5*01:01	DQB1*06:02	DQB1*06:04	DQA1*01:02		DPB1*02:01	DPB1*04:01	DPA1*01:03	
Sample16	Maternal	A*02:01	A*26:01	B*07:02	B*44:03	C*07:02	C*16:01	DRB1*01:01	DRB1*07:01	DRB4*01:01		DQB1*02:02	DQB1*05:01	DQA1*01:01	DQA1*02:01	DPB1*04:02	DPB1*10:01	DPA1*01:03	DPA1*02:01
	Fetal	A*03:01	A*26:01	B*07:02	B*35:01	C*04:01	C*07:02	DRB1*01:01				DQB1*05:01		DQA1*01:01		DPB1*04:02	DPB1*10:01	DPA1*01:03	DPA1*02:01
Sample17	Maternal	A*02:01	A*24:02	B*40:01		C*03:04		DRB1*13:02		DRB3*03:01		DQB1*06:04		DQA1*01:02		DPB1*02:01	DPB1*04:01	DPA1*01:03	
	Fetal	A*02:01	A*32:01	B*40:01		C*03:04		DRB1*04:04	DRB1*13:02	DRB3*03:01	DRB4*01:03	DQB1*03:02	DQB1*06:04	DQA1*01:02	DQA1*03:01	DPB1*04:01		DPA1*01:03	
Sample18	Maternal	A*02:05	A*03:01	B*44:02	B*49:01	C*05:01	C*07:01	DRB1*11:02	DRB1*13:01	DRB3*01:01	DRB3*02:02	DQB1*03:19	DQB1*06:03	DQA1*01:03	DQA1*05:05	DPB1*04:02	DPB1*15:01	DPA1*01:03	
	Fetal	A*02:01	A*02:05	B*13:02	B*49:01	C*06:02	C*07:01	DRB1*07:01	DRB1*11:02	DRB3*02:02	DRB4*01:03	DQB1*02:02	DQB1*03:19	DQA1*02:01	DQA1*05:05	DPB1*04:01		DPA1*01:03	
Sample19	Maternal	A*01:01	A*02:01	B*08:01	B*51:01	C*07:01	C*16:02	DRB1*03:01	DRB1*07:01	DRB3*01:01	DRB4*01:01	DQB1*02:01	DQB1*02:02	DQA1*02:01	DQA1*05:01	DPB1*02:01	DPB1*04:01	DPA1*01:03	
	Fetal	A*01:01	A*03:01	B*08:01	B*51:01	C*07:01	C*12:03	DRB1*03:01	DRB1*11:04	DRB3*01:01	DRB3*02:02	DQB1*02:01	DQB1*03:01	DQA1*05:01	DQA1*05:05	DPB1*01:01	DPB1*04:02	DPA1*01:03	DPA1*02:01
Sample20	Maternal	A*02:01	A*11:01	B*15:01	B*35:01	C*03:04	C*04:01	DRB1*04:07	DRB1*14:54	DRB3*02:02	DRB4*01:03	DQB1*03:01	DQB1*05:03	DQA1*01:04	DQA1*03:03	DPB1*03:01	DPB1*13:01	DPA1*01:03	DPA1*02:01
	Fetal	A*02:01	A*11:01	B*14:01	B*55:01	C*03:03	C*08:02	DRB1*03:01	DRB1*14:54	DRB3*01:01	DRB3*02:02	DQB1*02:01	DQB1*05:03	DQA1*01:04	DQA1*05:01	DPB1*04:02	DPB1*20:01	DPA1*01:03	
Sample21	Maternal	A*01:01	A*02:01	B*51:01	B*51:07	C*14:02		DRB1*04:03	DRB1*11:04	DRB3*02:02	DRB4*01:03	DQB1*03:01	DQB1*03:05	DQA1*03:01	DQA1*05:05	DPB1*04:01	DPB1*04:02	DPA1*01:03	
	Fetal	A*02:01	A*02:06	B*27:02	B*39:01	C*02:02	C*07:02	DRB1*14:05	DRB1*16:01	DRB3*02:02	DRB5*02:02	DQB1*05:02	DQB1*05:03	DQA1*01:02	DQA1*01:04	DPB1*04:01	DPB1*05:01	DPA1*01:03	DPA1*02:02
Sample22	Maternal	A*03:01	A*31:01	B*44:03	B*44:27	C*07:04	C*16:01	DRB1*07:01	DRB1*16:01	DRB4*01:01	DRB5*02:02	DQB1*02:02	DQB1*05:02	DQA1*01:02	DQA1*02:01	DPB1*04:01	DPB1*11:01	DPA1*01:03	DPA1*02:01
	Fetal	A*02:01		B*44:02	B*55:01	C*05:01	C*07:01	DRB1*14:54	DRB1*15:01	DRB3*02:02	DRB5*01:01	DQB1*05:03	DQB1*06:02	DQA1*01:02	DQA1*01:04	DPB1*02:01	DPB1*04:01	DPA1*01:03	
Sample23	Maternal	A*01:01	A*29:02	B*08:01	B*44:03	C*07:01	C*16:01	DRB1*03:01	DRB1*07:01	DRB3*01:01	DRB4*01:01	DQB1*02:01	DQB1*02:02	DQA1*02:01	DQA1*05:01	DPB1*02:01	DPB1*11:01	DPA1*01:03	DPA1*02:01

	Fetal	A*24:02		B*07:02	B*44:02	C*07:02	C*16:04	DRB1*11:04	DRB1*15:01	DRB3*02:02	DRB5*01:01	DQB1*03:01	DQB1*06:02	DQA1*01:02	DQA1*05:05	DPB1*04:01		DPA1*01:03	
Sample24	Maternal	A*01:01	A*33:01	B*18:01	B*37:01	C*05:01	C*06:02	DRB1*07:01	DRB1*16:01	DRB4*01:01	DRB5*02:02	DQB1*02:02	DQB1*05:02	DQA1*01:02	DQA1*02:01	DPB1*15:01	DPB1*17:01	DPA1*01:03	DPA1*02:01
	Fetal	A*30:01	A*33:01	B*42:01	B*78:01	C*16:01	C*17:01	DRB1*08:04	DRB1*11:02	DRB3*02:02		DQB1*03:01	DQB1*03:19	DQA1*05:05		DPB1*01:01	DPB1*29:01	DPA1*01:03	DPA1*02:01
Sample25	Maternal	A*02:01	A*02:01	B*13:02	B*15:01	C*03:04	C*06:02	DRB1*04:01	DRB1*07:01	DRB4*01:03		DQB1*02:02	DQB1*03:02	DQA1*02:01	DQA1*03:01	DPB1*04:01	DPB1*17:01	DPA1*01:03	DPA1*02:01
	Fetal	A*01:01	A*03:01	B*07:02	B*08:01	C*07:01	C*07:02	DRB1*13:01		DRB3*01:01	DRB3*02:02	DQB1*06:03		DQA1*01:03		DPB1*04:01	DPB1*09:01	DPA1*01:03	DPA1*02:01
Sample26	Maternal	A*02:06	A*31:01	B*08:01	B*27:05	C*03:03	C*07:02	DRB1*04:01	DRB1*08:01	DRB4*01:03		DQB1*03:01	DQB1*04:02	DQA1*03:03	DQA1*04:01	DPB1*04:01	DPB1*04:02	DPA1*01:03	
	Fetal	A*01:01		B*08:01	B*08:01	C*07:01	C*07:02	DRB1*03:01	DRB1*15:01	DRB3*02:02	DRB5*01:01	DQB1*02:01	DQB1*06:02	DQA1*01:02	DQA1*05:01	DPB1*04:01		DPA1*01:03	
Sample27	Maternal	A*03:01	A*32:01	B*44:02	B*57:01	C*06:02	C*07:04	DRB1*07:01	DRB1*14:60	DRB3*02:02	DRB4*01:03N	DQB1*03:03	DQB1*05:03	DQA1*01:04	DQA1*02:01	DPB1*01:01	DPB1*13:01	DPA1*02:01	DPA1*02:01
	Fetal	A*01:01	A*24:02	B*18:01	B*40:01	C*03:04	C*07:01	DRB1*04:04	DRB1*13:02	DRB3*03:01	DRB4*01:03	DQB1*03:02	DQB1*06:04	DQA1*01:02	DQA1*03:01	DPB1*03:01		DPA1*01:03	

Table S7. Sequences of primers used for real-time quantitative PCR.

	Forward primer	Reverse primer	Amplicon size (bp)
CXCL8	GAAGGAACCATCTCACTG	CCACTCTCAATCACTCTC	200
IL-1β	TGGCTTATTACAGTGGCAATG	GTGGTGGTCGGAGATTCG	134
TNFα	CCCCAGGGACCTCTCTCTAATC	TACAACATGGGCTACAGGCTTG	91
CXCL2	TCACAGTGTGTGGTCAACAT	AACACAGAGGGAAACACTGC	139
IL-6	TGAGAGTAGTGAGGAACAAG	CGCAGAATGAGATGAGTTG	188
TGF-β1	CCCAGCATCTGCAAAGCTC	GTCAATGTACAGCTGCCGCA	101
ACTB	ACCACACCTTCTACAATGAG	TAGCACAGCCTGGATAGC	161
GAPDH	ACCCACTCCTCCACCTTTGAC	TCCACCACCCTGTTGCTGTAG	110
HPRT1	AGATGGTCAAGGTCGCAAGC	TCAAGGGCATATCCTACAACAAAC	115
RPL13A	CCTGGAGGAGAAGAGGAAAGAGA	TTGAGGACCTCTGTGTATTTGTCAA	126
HMBS	GGCAATGCGGCTGCAA	GGGTACCCACGCGAATCAC	64
